# αM-Conotoxin MIIIJ Blocks Nicotinic Acetylcholine Receptors at Neuromuscular Junctions of Frog and Fish

**DOI:** 10.3390/toxins12030197

**Published:** 2020-03-21

**Authors:** Matthew J. Rybin, Henrik O’Brien, Iris Bea L. Ramiro, Layla Azam, J. Michael McIntosh, Baldomero M. Olivera, Helena Safavi-Hemami, Doju Yoshikami

**Affiliations:** 1 Department of Biology, University of Utah, Salt Lake City, UT 84112, USA; mxr2011@miami.edu (M.J.R.); henrikobrien@gmail.com (H.O.); iris.ramiro@sund.ku.dk (I.B.L.R.); layla.azam@utah.edu (L.A.); michael.mcintosh@utah.edu (J.M.M.); olivera@biology.utah.edu (B.M.O.); 2 Department of Psychiatry and Behavioral Sciences, University of Miami, Miller School of Medicine, Miami, FL 33136, USA; 3 Department of Biomedical Sciences, University of Copenhagen, 2200 Copenhagen, Denmark; 4 George E. Whalen Veterans Affairs Medical Center, Salt Lake City, UT 84112, USA; 5 Department of Psychiatry, University of Utah, Salt Lake City, UT 84112, USA; 6 Department of Biochemistry, University of Utah, Salt Lake City, UT 84112, USA

**Keywords:** venom, conotoxin, nicotinic acetylcholine receptor, neuromuscular junction

## Abstract

We report the discovery and functional characterization of αM-Conotoxin MIIIJ, a peptide from the venom of the fish-hunting cone snail *Conus magus*. Injections of αM-MIIIJ induced paralysis in goldfish (*Carassius auratus*) but not mice. Intracellular recording from skeletal muscles of fish (*C. auratus*) and frog (*Xenopus laevis*) revealed that αM-MIIIJ inhibited postsynaptic nicotinic acetylcholine receptors (nAChRs) with an IC_50_ of ~0.1 μM. With comparable potency, αM-MIIIJ reversibly blocked ACh-gated currents (I_ACh_) of voltage-clamped *X. laevis* oocytes exogenously expressing nAChRs cloned from zebrafish (*Danio rerio*) muscle. αM-MIIIJ also protected against slowly-reversible block of I_ACh_ by α-bungarotoxin (α-BgTX, a snake neurotoxin) and α-conotoxin EI (α-EI, from *Conus ermineus* another fish hunter) that competitively block nAChRs at the ACh binding site. Furthermore, assessment by fluorescence microscopy showed that αM-MIIIJ inhibited the binding of fluorescently-tagged α-BgTX at neuromuscular junctions of *X. laevis,* *C. auratus*, and *D. rerio*. (Note, we observed that αM-MIIIJ can block adult mouse and human muscle nAChRs exogenously expressed in *X. laevis* oocytes, but with IC_50_s ~100-times higher than those of zebrafish nAChRs.) Taken together, these results indicate that αM-MIIIJ inhibits muscle nAChRs and furthermore apparently does so by interfering with the binding of ACh to its receptor. Comparative alignments with homologous sequences identified in other fish hunters revealed that αM-MIIIJ defines a new class of muscle nAChR inhibitors from cone snails.

## 1. Introduction

Cone snail venoms have a profusion of peptides that target ion channels of the nervous system [1,2,3]. Owing to their diversity and target specificity, cone snail toxins (conotoxins) have become valuable tools for ion channel research and drug design [3,4,5]. Cone snails form a diverse genus of ~800 extant species that have evolved diverse diets and predation strategies. *Conus magus* belongs to one of the most studied clades of fish-hunting cone snails, the *Pionoconus* clade. *Pionoconus* cone snails use the taser-and-tether hunting strategy that is characterized by rapid, irreversible paralysis following venom injection [6]. This is achieved by the concerted action of toxins that target ion channels and receptors of the peripheral nervous system, particularly those at the neuromuscular junction [7]. Many of the paralytic components of *Pionoconus* venom have been characterized. For example, the venom of the fish hunter *Conus magus* has three families of paralytic peptides, and a representative member of each family is as follows: α-conotoxin MI, which blocks postsynaptic nicotinic acetylcholine receptors (nAChRs) that are essential for excitatory postsynaptic potentials (EPSPs) in muscle fibers; ω-conotoxin MVIIA, which blocks voltage-gated calcium channels (V-gated Ca channels) whose activity triggers transmitter (acetylcholine, ACh) release from the presynaptic motor-nerve terminal; and μ-conotoxin MIIIA, which blocks voltage-gated sodium channels (V-gated Na channels) responsible for action potentials in muscle fibers (see Table 1 for peptide sequences and references). Here, we describe the identification of αM-MIIIJ, the first member of a previously uncharacterized family of paralytic toxins with functional but no structural similarity to α-conotoxin MI.

αM-MIIIJ was originally identified by fractionation of *C. magus* venom and sequencing of some of its major components during the purification and characterization of α-MI by McIntosh et al. in 1982 [8]. Because αM-MIIIJ did not elicit a visible phenotype upon intracranial injections into mice and was inactive in blocking synaptic transmission at the neuromuscular junction of the grass frog, *Rana pipiens*, the pharmacological characterization of this toxin was not further pursued, and the discovery of this peptide not reported.

Here, using electrophysiological experiments, we demonstrate that αM-MIIIJ blocks the function of nAChRs at the neuromuscular junctions (NMJs) of African clawed frog (*Xenopus laevis*) and goldfish (*Carassius auratus*). We confirm that αM-MIIIJ does not block nAChRs at the NMJ of *R. pipiens*. We also show that αM-MIIIJ reversibly blocks muscle nAChRs cloned from zebrafish (*Danio rerio*) and exogenously expressed in *X. laevis* oocytes; moreover, αM-MIIIJ protects these receptors from block by α-bungarotoxin (α-BgTX) and α-conotoxin EI (α-EI). We believe this is the first report where an nAChR-targeting conotoxin from a fish hunter has been functionally tested on molecularly-defined fish nAChRs. Finally, we show by fluorescence microscopy that αM-MIIIJ inhibits the binding of fluorescently-labeled α-BgTX to nAChRs at NMJs of frog (*X. laevis*, but not *R. pipiens*) and both goldfish and zebrafish. Mining of published transcriptome data of *C. magus* and related cone snail species suggests that αM-MIIIJ is the first functionally characterized member of a large class of toxins expressed in taser-and-tether cone snails.

## 2. Results

### 2.1. αM-MIIIJ Discovery

**Figure 1:**
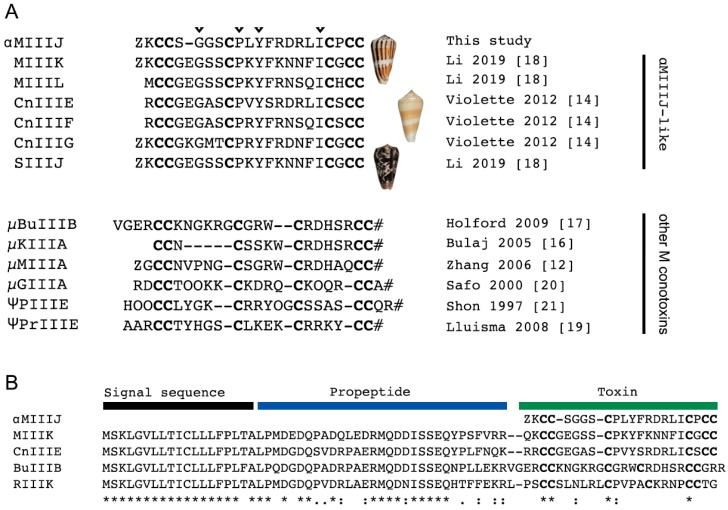
Sequence of αM-MIIIJ compared with those of other conotoxins. **A.** Sequence alignment of αM-MIIIJ with related sequences of unknown activity identified in *C. magus*, *C. consors*, and *C. striatus* (top) and M-superfamily conotoxins (bottom) that block V-gated Na channels (μ-prefix) or nAChRs (ψ-prefix for binding at a noncompetitive site). Cysteines are in bold. Identical amino acids are shown with arrowhead on top of αM-MIIIJ sequence. Z: pyroglutamic acid, O: hydroxyproline, #: C-terminal amidation. References [12,14,15,16,17,18,19,20]. **B.** Precursor sequence alignment of M-superfamily toxins highlights a conserved signal sequence used for toxin gene classification. The precursor sequence of αM-MIIIJ could not be retrieved, but high sequence similarities with αM-MIIIJ-like sequences, including MIIIK (91%), strongly suggest that αM-MIIIJ also belongs to the M-superfamily. Amino acid conservations are denoted by an asterisk (*). Full stops (.) and colons (:) represent a low and high degree of similarity, respectively.

αM-MIIIJ was discovered, albeit not reported, during our initial purification of α-MI [8]. Upon reversed-phase fractionation of crude venom from *C. magus*, αM-MIIIJ eluted considerably later than α-MI (Figure S1). Further purification and sequencing of the reversed-phase fraction led to the structural characterization of αM-MIIIJ. αM-MIIIJ has 22 amino acids including an N-terminal pyro-glutamate (Figure 1A), and a molecular mass of 2429 Da (see below). Furthermore, αM-MIIIJ has six cysteines connected by three disulfide bonds and exhibits a type III cysteine framework (CC-C-C-CC) [13]. Mining of the venom gland transcriptome of *C. magus* and two other species from the *Pionoconus* clade, *Conus consors* and *Conus striatus*, identified several toxins with high sequence similarity to αM-MIIIJ (Figure 1A). Of these, the *C. consors* peptides (CnIIIE, CnIIIF, CnIIIG) were previously shown to be part of the injected venom cocktail of this species, but their activity was not explored [14]. 

According to their conserved N-terminal signal sequence, conotoxins can be grouped into genetically related toxin superfamilies [3]. While we were not able to retrieve the precursor sequence of αM-MIIIJ, the full-length precursors of highly similar sequences could be unambiguously assigned to the M-superfamily (Figure 1B) [21] strongly suggesting that αM-MIIIJ is also a member of the M-superfamily. For example, αM-MIIIJ and MIIIK share 74% sequence identity and 91% sequence similarity. The absence of an identical sequence to αM-MIIIJ in the published transcriptome data of *C. magus* can be attributed to the high intraspecies sequence variation of cone snail toxins. The venom used in our study was not extracted from the same specimen as that used for transcriptome sequencing. Thus, we did not necessarily anticipate finding an exact sequence match but believe that the difference in amino acid sequence is due to intraspecies variation. αM-MIIIJ was named according to the conventional conotoxin nomenclature, where the Greek letter represents the pharmacological class (“α” for inhibitors of nAChRs), followed by a single letter for the gene family (“M” for M-superfamily), one or two letters representing the species name (“M” for *magus*), a Roman numeral for the cysteine framework (“III”) and a letter or number given to identify different toxins belonging to the same class (“J”).

### 2.2. αM-MIIIJ Synthesis

Solid-phase synthesis was carried out as described in Methods. The final product showed 98% purity by analytical HPLC. The observed monoisotopic (M + H)^+^ value of the synthetic peptide was 2428.6, which corresponded with the calculated (M + H)^+^ value of 2429.0. The peptide was co-injected with the venom-purified peptide and analyzed by capillary zone electrophoresis (CZE); there was no difference in the migration between the native and the synthetic peptide. 

### 2.3. Paralytic Effects of αM-MIIIJ

In-vivo assays revealed that αM-MIIIJ paralyzed goldfish (Table S1) but not mice. Three Swiss Webster mice (22.34, 8.65, 8.52 g) were injected intraperitoneally (i.p.) with αM-MIIIJ (~2.3 nmol/g mouse) and observed without any reduced motor function or paralysis for >1.5 h. For comparison, a mouse (8.28 g) was injected i.p. with α-conotoxin EI (α-EI, 0.24 nmol/g mouse), an antagonist of muscle nAChRs [22], that caused paralysis and then death in <10 min. This initial observation suggested muscle nAChR may not be the molecular target of αM-MIIIJ, at least not in mice. To identify its molecular target, we examined the effect of αM-MIIIJ on isolated muscle preparations from lower vertebrates, namely two species each of frog and fish as well as on cloned muscle nAChRs of zebrafish exogenously expressed in *X. laevis* oocytes. 

### 2.4. Extracellular Electrophysiology of Frog Muscle Preparations

**Figure 2:**
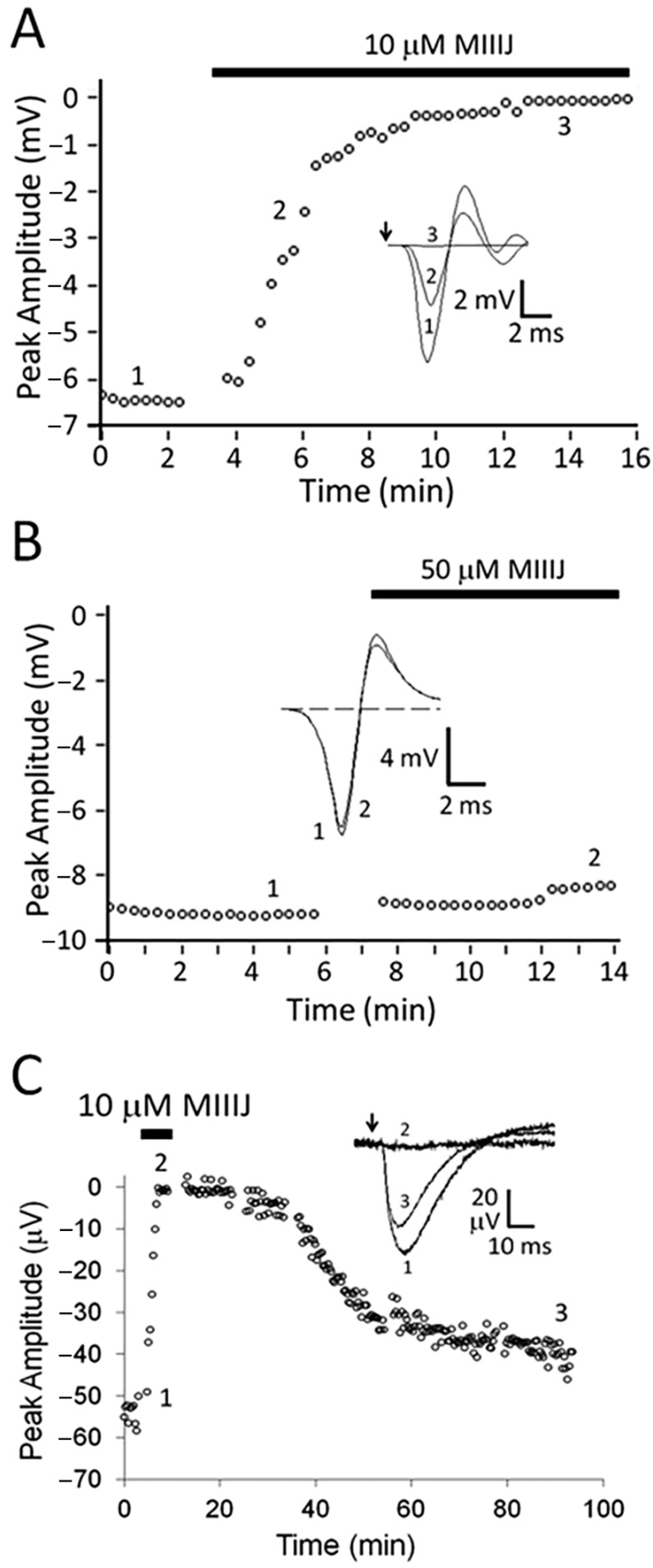
αM-MIIIJ blocks excitatory synaptic responses but not action potentials in *X. laevis* longitudinal pectoralis (LP) muscle. Extracellular recording from adult muscle was performed as described in Methods. Graphs plot peak amplitudes of responses versus time, where presence of αM-MIIIJ is represented by black bar above each plot and insets show representative traces at the enumerated time points. Arrows in insets of panels A and C show when stimulus was applied. A and B. Muscle action potentials in response to indirect (**A**) and direct (**B**) stimulation. Ten μM αM-MIIIJ completely blocked action potentials evoked by nerve stimulation (**A**); in contrast, a five-times higher concentration of αM-MIIIJ had essentially no effect on action potentials evoked by direct stimulation (**B**). As a positive control, μ-PIIIA (10 μM), a conotoxin that irreversibly inhibits muscle V-gated Na channels, completely blocked action potentials within 10 min. (flat dashed trace of panel B’s inset). **C**. Motor-nerve stimulation evoked synaptic responses (without confounding muscle action potentials) in muscle preparation treated with μ-PIIIA (10 μM). The synaptic responses were reversibly blocked by 10 μM αM-MIIIJ. Each trace in inset of panel C represents the average of ~10 responses. Results replicating those illustrated here (i.e., complete block by toxin of nerve stimulation-evoked action potentials and synaptic responses, and conversely no block by toxin of direct stimulation-evoked action potentials) were obtained in three other LP muscle preparations.

αM-MIIIJ readily blocked extracellularly-recorded action potentials in *X. laevis* longitudinal pectoralis (LP) muscle evoked by motor-nerve stimulation (Figure 2A) but not those evoked by direct muscle stimulation (Figure 2B). The resistance of directly-evoked action potentials to αM-MIIIJ indicates that V-gated Na channels in the muscle were not blocked by the peptide. To examine the susceptibility of synaptic potentials to the peptide, preparations were treated with μ-PIIIA, a conotoxin that irreversibly blocks V-gated Na channels in frog muscle [23]; thus, only synaptic potentials are observed upon nerve stimulation. Such synaptic potentials were readily blocked by 10 μM αM-MIIIJ (Figure 2C). This result suggests that αM-MIIIJ blocked a target that directly mediated synaptic transmission; for example, either a presynaptic voltage-gated calcium channel or postsynaptic nAChR. 

**Figure 3:**
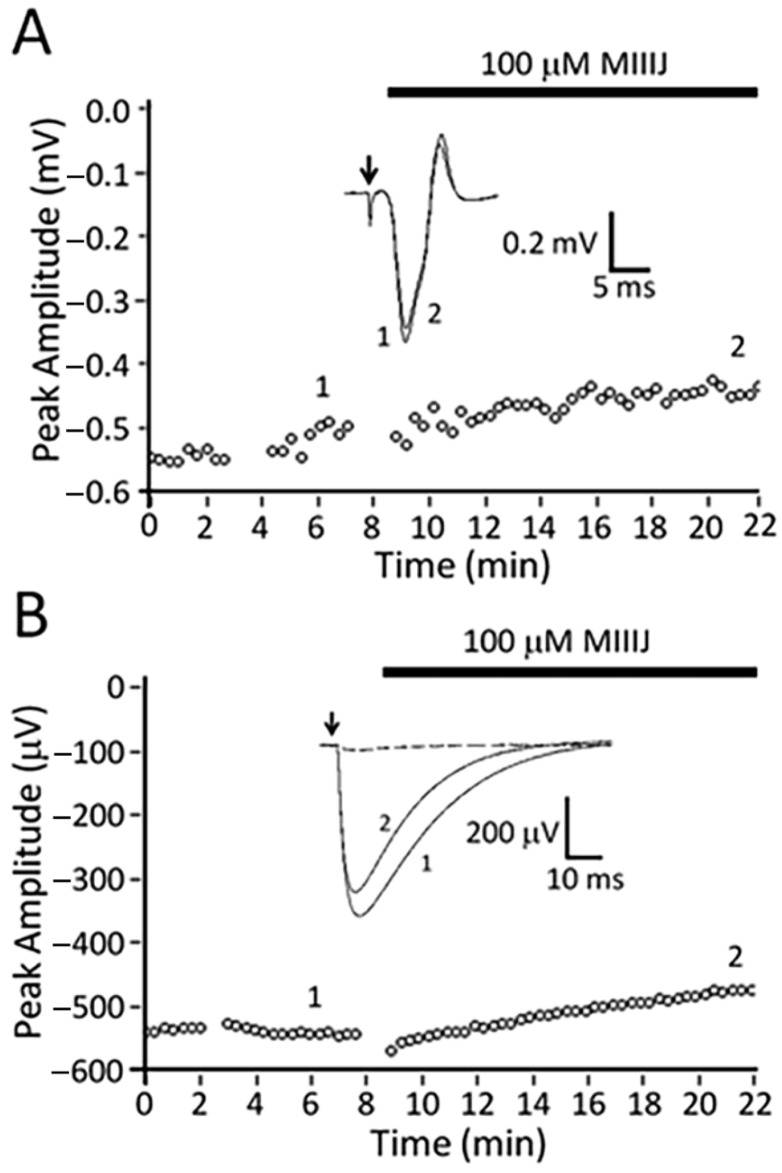
Action and synaptic potentials in *R. pipiens* cutaneous pectoralis (CP) muscle are unaffected by αM-MIIIJ (100 μM). Muscle preparation and recording were as described in Methods. Format of presentation of results are as in Figure 2 (except traces in panel B were acquired with a low pass-filter setting of 0.1, instead of 1, Hz). Indirectly-evoked action potentials (**A**) and synaptic responses (**B**) are essentially unaffected by 100 μM αM-MIIIJ. In B, the muscle was treated with μ-PIIIA as in Figure 2C. As a positive control, the preparation was exposed to the muscle nAChR antagonist d-tubocurare (10 μM), which blocked within 15 min. (largely flat, dashed trace of panel B’s inset). These results indicate that αM-MIIIJ does not block the muscle action potential in *R. pipiens* (like in *X. laevis*) muscle, nor does αM-MIIIJ block (unlike in *X. laevis*) the synaptically-evoked response. Results replicating those illustrated here (i.e., no block of either action or synaptic potentials by toxin) were obtained in three other CP muscle preparations.

When *R. pipiens* muscles were tested, αM-MIIIJ did not block responses evoked by indirect stimulation (Figure 3). The persistence of the muscle action potential (Figure 3A) meant that V-gated Na channels were not blocked; moreover, this result also implied that the underlying synaptic response that triggered the action potential was also insensitive to αM-MIIIJ. The latter implication was examined more closely by looking directly at synaptic responses in muscles pre-treated with μ-PIIIA. Indeed, synaptic potentials in *R. pipiens* muscle were resistant to block by αM-MIIIJ even at a 10-fold higher concentration than that which rapidly obliterated synaptic potentials in *X. laevis* muscle (compare Figure 3B with Figure 2C).

**Figure 4:**
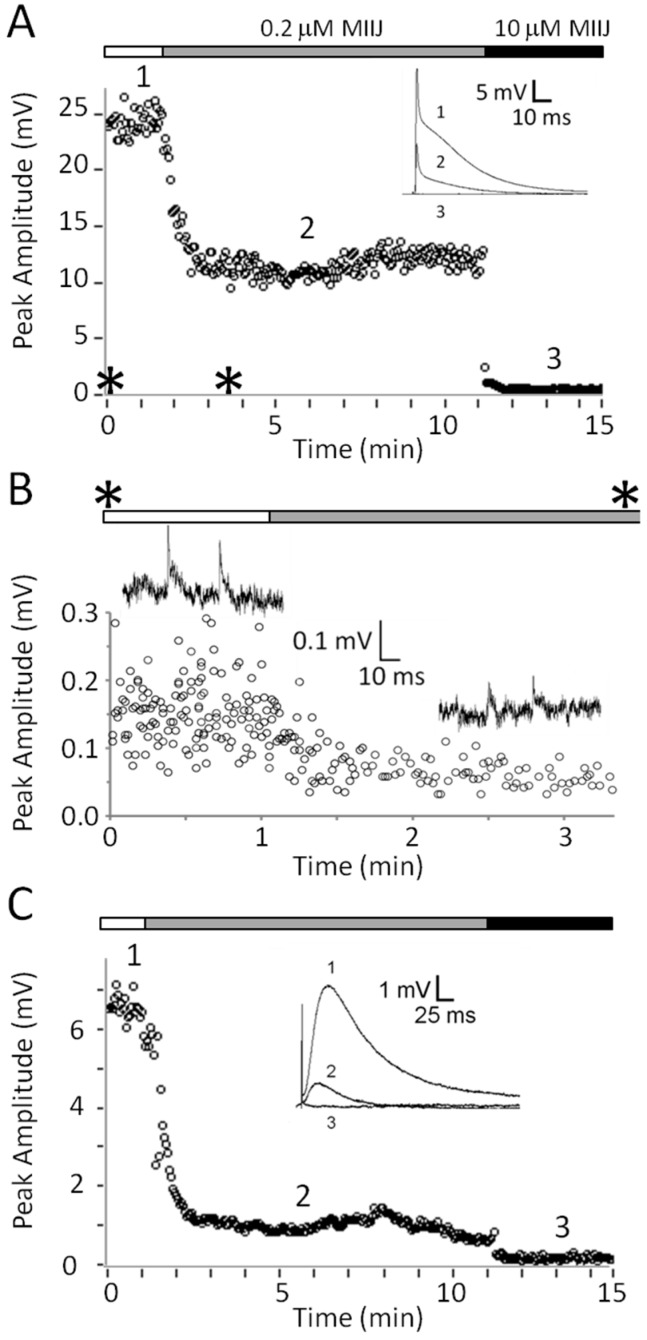
αM-MIIIJ blocks excitatory postsynaptic potentials (EPSPs), miniature excitatory postsynaptic potentials (MEPSPs), and ACh-evoked postsynaptic potentials (PSPs) in *X. laevis* LP muscle. Intracellular recording from juvenile muscle preparation was performed as described in Methods. All responses were obtained contemporaneously from one muscle fiber whose resting potential ranged between −77 to −88 mV. A–C. Time course of block by 0.2 or 10 μM αM-MIIIJ of EPSPs (**A**), MEPSPs (**B**), and ACh-evoked PSPs (**C**). The pair of asterisks along the top of panel B denotes the time interval flanked by the pair of asterisks in panel A. Insets in panels A and C show sample traces before (1) and in the presence of 0.2 μM αM-MIIIJ (2) or 10 μM αM-MIIIJ (3). Hump in falling phase of EPSPs in inset of panel A presumably reflects the multiple innervation of the fiber. Sample traces in panel B were obtained before (left) and in the presence of 0.2 μM αM-MIIIJ (right); each trace shows two MEPSPs, and the calibration scale applies to both traces. No MEPSPs were discernable in the presence of 10 µM αM-MIIIJ. Early spike of superimposed traces in inset of C is an artifact of the iontophoretic pulse.

To examine whether synaptic block by αM-MIIIJ in the *X. laevis* preparation was pre- or post-synaptic, intracellular recordings were made from muscles pretreated with μ-PIIIA to abolish muscle twitches normally evoked by motor nerve stimulation. In addition, preparations were treated with fluorescein-peanut agglutinin (f-PNA) to locate NMJs to assist in the placement of the intracellular recording electrode and the ACh-iontophoresis electrode, as described in Methods. αM-MIIIJ blocked nerve-stimulation evoked EPSPs (Figure 4A), spontaneous miniature EPSPs (MEPSPs, Figure 4B) and responses to iontophoreticaly-applied ACh (Figure 4C). αM-MIIIJ blocked these responses >50% at a concentration of 0.2 μM, and 100% at a concentration of 10 μM. Block of EPSPs, MEPSPs and ACh-evoked reponses was also observed at other αM-MIIIJ concentrations on separate muscle preparations (see Figure 6). These results clearly show that αM-MIIIJ blocks nAChRs in *X. laevis* muscle.

### 2.5. Intracellular Recording of Synaptic Activity in Goldfish Intercostal (IC) Muscle

**Figure 5:**
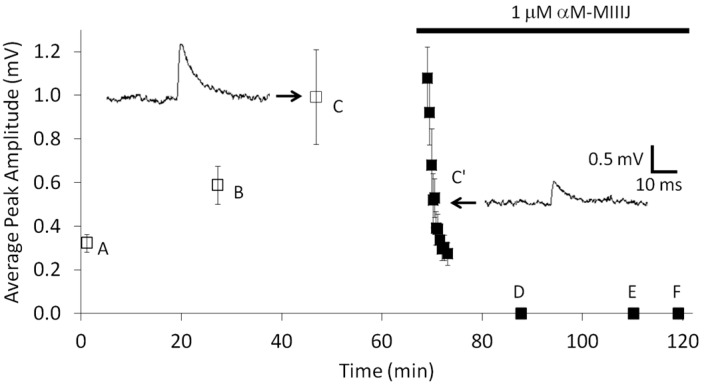
αM-MIIIJ (10 μM) blocks MEPSPs in goldfish intercostal (IC) muscles. Intracellular recordings, performed as described in Methods, were obtained successively from six muscle fibers, A through F. Each data point represents the mean peak amplitude ± SD of at least 30 events. Recordings were made before (fibers A–C) and during (fibers C’–F) exposure to αM-MIIIJ (1 μM). After recordings from fiber C were acquired, the bath was supplemented with a high concentration of αM-MIIIJ such that its final concentration in the bath was 1 μM, and the recording recommenced (fiber C’) until the resting potential was abruptly lost. The resting potentials (in mV) of the respective fibers fibers were as follows: −67 (fiber A), −62 (fiber B), −92 (fiber C), −84 (fiber C’), −98 (fiber D), −65 (fiber E), and −101 (fiber F). In fibers D–F, no discernable MEPSPs were observed above the noise and indicated the block by 1 μM αM-MIIIJ was > 80% in each case.

In view of αM-MIIIJ’s ability to paralyze goldfish as well as block nAChRs in *X. laevis* frog, we examined whether the peptide also blocked nAChR function in goldfish, whose intercostal (IC) muscle proved simple to dissect (see Section 5.6.). Spontaneous MEPSPs were readily observed regardless of the site of penetration of the intracellular recording electrode. This is because the muscle fibers are relatively short (~1 mm) and have NMJs distributed all over their surface (see Figure S2B) as expected of fish fast muscles. αM-MIIIJ (1 μM) readily blocked spontaneous MEPSPs in goldfish IC muscles (Figure 5).

**Figure 6:**
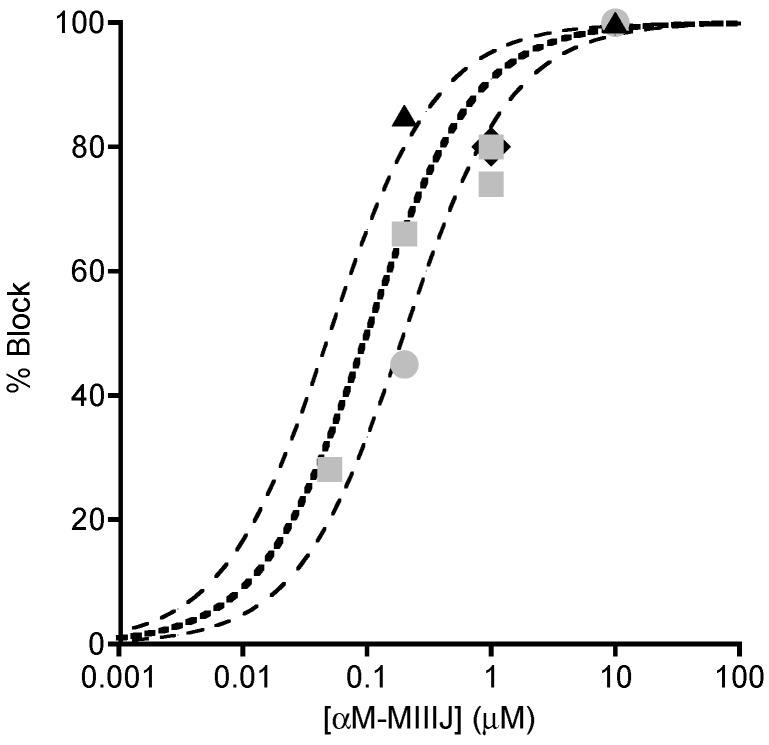
Concentration-dependent block by αM-MIIIJ of synaptically- and ACh-evoked responses at frog and fish NMJs. Plot summarizing results with different concentrations of αM-MIIIJ from experiments of the sort illustrated in Figure 4; Figure 5. Percentage block of: EPSPs in *X. laevis* LP (circles, 2); spontaneous MEPSPs in *X. laevis* LP (squares, 4) and goldfish IC (diamond, which denotes the minimum block of 80% by 1 μM peptide in fibers D, E, and F in Figure 5); and ACh-evoked responses in *X. laevis* LP (triangles, 2). Each point represents one measurement. Block of EPSPs were not corrected for non-linear summation [24] and are therefore minimum estimates. Lines are curves of the Langmuir adsorption isotherm, % Block = 100%/{1 + (IC_50_/[Toxin])}, where [Toxin] is αM-MIIIJ concentration, and IC_50_ = 0.1, 0.05 or 0.2 μM (central bold-dotted line and flanking dashed lines, respectively). Experimental points lie close to or between the dashed lines and suggest an aggregate IC_50_ within a factor of about two of 0.1 μM.

αM-MIIIJ clearly blocks the ACh-mediated postsynaptic responses in both *X. laevis* and goldfish muscles. The block was concentration dependent as illustrated in Figure 6, which combines the results from different preparations. In the figure, there are also three binding curves for a simple bimolecular interaction of toxin and receptor with an IC_50_ of 0.05, 0.1 or 0.2 μM (see Figure 6 legend for equation). The potencies of αM-MIIIJ in blocking nACh receptors of *X. laevis* and goldfish muscles were similar, with an IC_50_ estimated to be within about a factor of two of 0.1 μM.

### 2.6. αM-MIIIJ Reversibly Blocks ACh-Gated Currents (I_ACh_) of Zebrafish-Muscle nAChRs Exogenously Expressed in X. laevis Oocytes

**Figure 7:**
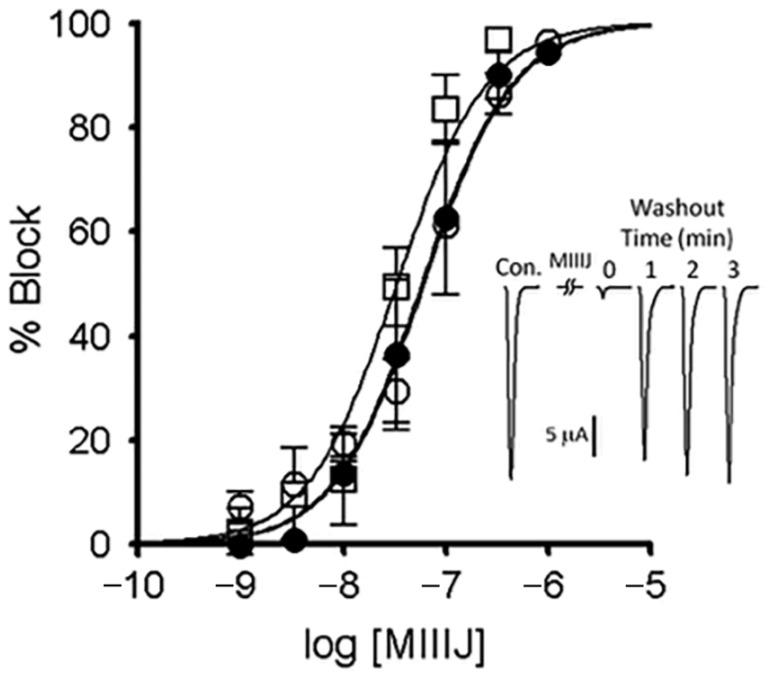
αM-MIIIJ blocks ACh-gated currents (I_ACh_) of voltage-clamped *X. laevis* oocytes exogenously expressing zebrafish nAChRs. Expression of nAChRs, induction of I_ACh_ in voltage-clamped oocytes, and toxin application and analysis of consequent effects were performed as described in Methods. Concentration-dependent block by αM-MIIIJ of three combinations of zebrafish nAChR subunits are plotted (αβδε, closed circles; αβδγ, open circles; and αβδ, open squares). Each data point represents mean ± SE (*n* = 3 or 4 oocytes). Solid line represents best-fit curve of the data to the Langmuir adsorption isotherm (see Table 2 for IC_50_s). Inset illustrates example I_ACh_ traces from an oocyte expressing the αβδ-subunit combination before (control), immediately after a 5-min. exposure to 330 nM αM-MIIJ, and following peptide washout at 1-min. intervals; duration of each trace is 4 sec. The block by αM-MIIIJ was readily reversible.

Three combinations of nAChRs subunits expressed in zebrafish muscle have been identified: αβδγ (embryonic fibers), αβδε (larval and adult fast fibers), and αβδ (slow fibers) [25] (see also [26,27]). The susceptibilities of these nAChRs to αM-MIIIJ were examined in two-electrode voltage-clamped oocytes expressing each of the three combinations of zebrafish subunits, and the ACh-gated currents (I_ACh_) were measured to monitor the function of the nAChRs. αM-MIIIJ blocked the I_ACh_ of all three subunit combinations with IC_50_s in the range of 50 nM (Figure 7, IC_50_ values are in Table 2). These results are consistent with what we observed with nAChRs endogenously expressed in muscles of *Xenopus* frog and goldfish (Figure 6).

**Figure 8:**
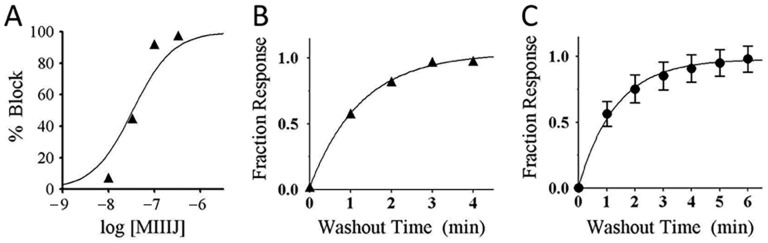
αM-MIIIA block of αβε nAChR (A), and washout kinetics from αβε AChR (B) and αβδε nAChR (C). These experiments were done essentially as in Figure 7. **A**. αM-MIIIJ concentration–response curve for block of αβε nAChR (see Table 2 for IC_50_). The data points follow a trajectory steeper than their fit to a Langmuir isotherm (solid curve), the significance of which remains to be determined. **B, C.** αM-MIIIJ washout curves from αβε (**B**) and αβδε (**C**) nAChRs. Solid line is the best-fit single-exponential curve with a k_off_ of 0.80 min^−1^ (95% C.I. of 0.7–0.9) for αβε, and 0.76 min^−1^ (95% C.I. of 0.4–1.2) for αβδε. Data points represent mean ± S.E. (*n* = 3 for panels **A** and **B**; and *n* = 6 for panel **C**, from aggregate of open circles in Figure 9B,E below).

nAChRs comprised of only three different subunit combinations (αβγ, αβδ, and αβε receptors) are not endogenously expressed except in zebrafish, which express αβδ nAChRs as described above. Such combinations of mammalian nAChR subunits have been exogenously expressed in *X. laevis* oocytes to help identify the binding sites of competitive nAChR antagonists (e.g., [29]). Toward that end, we also tried to functionally express zebrafish αβγ- and αβε-receptor combinations in oocytes. Thus far, we have successfully expressed only the latter and characterized its block by αM-MIIIJ (Figure 8A,B).

**Figure 9:**
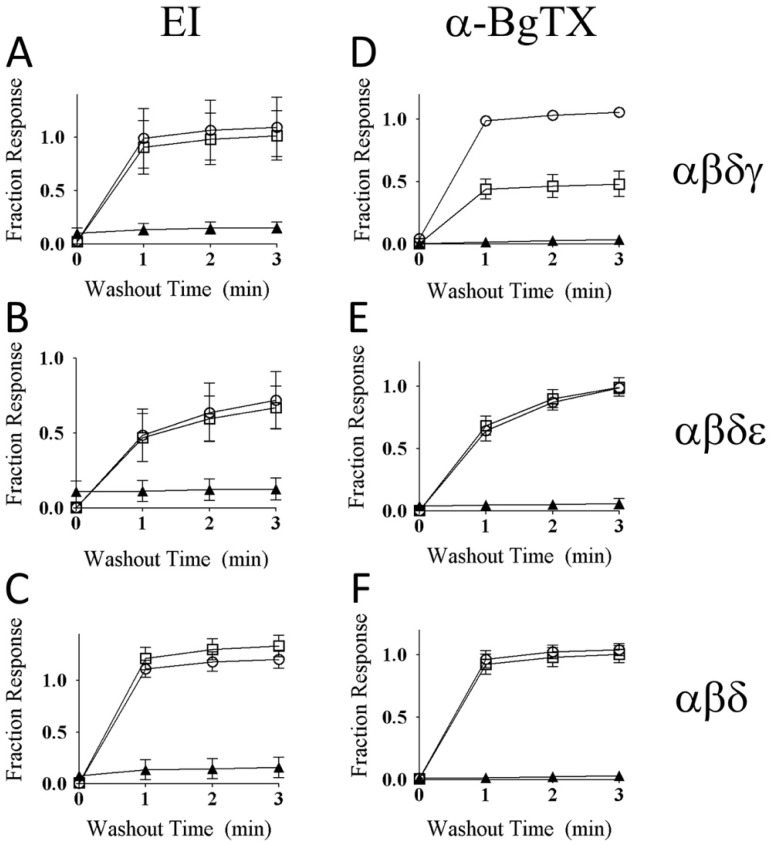
αM-MIIIJ (10 μM) protects zebrafish nAChRs against slowly-reversible block by α-EI and α-bungarotoxin (α-BgTX). ACh-gated currents from oocytes expressing zebrafish nAChRs were obtained as described in Methods. A given oocyte was first exposed to 10 μM αM-MIIIJ for 10 min. in a static bath, then perfused to observe the time course of recovery from block (open circles). The oocyte was then exposed again to 10 μM αM-MIIIJ for 5 min. in a static bath, after which the bath was supplemented with either 1 μM α-EI (open squares in panels A, B, and C) or 10 μg/mL α-BgTX (open squares in panels D, E, and F) and allowed to sit for another 5 min before the bath perfusion was recommenced. Finally, in a separate experiment, a given oocyte was exposed to either 1 μM α-EI alone (panels **A**, **B**, and **C**) or 10 μg/mL of α-BgTX alone (panels **D**, **E**, and **F**) for 5-min in a static bath before bath perfusion was recommenced (solid triangles). The time courses of recovery from block during the perfusion following the static-bath exposure to toxin(s) are plotted. Recovery from block following exposure to α-EI alone or α-BgTX alone was very slow in all panels (solid triangles). Furthermore, in all instances, except panel D, pre- and concurrent exposure to αM-MIIIJ prevented persistent block by α-EI and α-BgTX (as evident from similarity of time courses curves denoted by open circles and open squares). The divergent open-circle and open-square curves in panel D indicate that αM-MIIIJ only partially protected the αβδγ receptor against block by α-BgTX. α-BgTX use in these experiments was derivatized with tetramethylrhodamine (same stock solution as that used in fluorescence imaging experiments described below).

The reversibility of αM-MIIIJ’s block of I_ACh_ during its washout from αβδε and αβε nAChRs was relatively slow (k_off_ of 0.80 min^−1^ (95% C.I. of 0.7–0.9) for αβε, and 0.76 min^−1^ (95% C.I. of 0.4–1.2) for αβδε, Figure 8B,C). For subunit combinations lacking the ε-subunit (αβδ and αβδγ), however, I_ACh_ recovered almost completely within a minute of αM-MIIIJ washout (e.g., Figure 7 inset) with a k_off_ of 2.4 min^−1^ (95% C.I. of 0.0–5.2) for αβδγ, and 2.5 min^−1^ (95% C.I. of 1.1–3.9) for αβδ (data were the aggregate of open circles in Figure 9A,D and C,F, respectively).

### 2.7. αM-MIIIJ Protects Zebrafish nAChRs Against A-Bungarotoxin (α-BgTX) and A-Conotoxin EI (α-EI)

**Figure 10:**
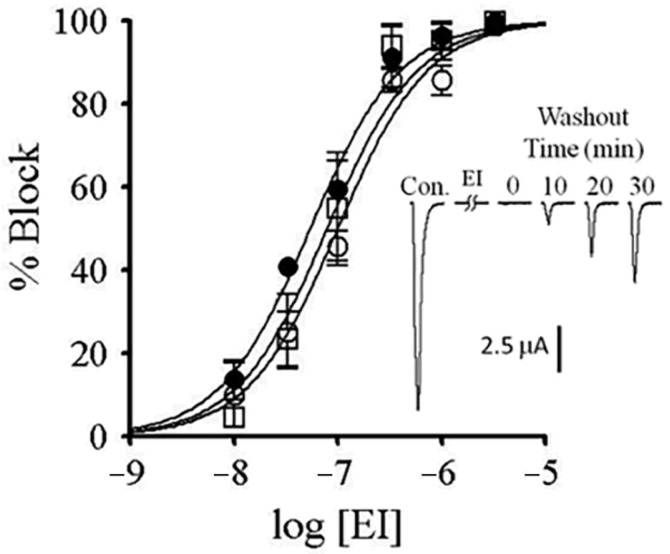
Block by α-conotoxin EI of zebrafish muscle nAChRs expressed in *X. laevis* oocytes. The block of I_ACh_ by conotoxin was assessed as described in Figure 7. Plotted are the concentration–response curves for the block by α-EI of αβδ (open squares), αβδγ (open circles), and αβδε (closed circles) nAChRs. Each data point represents mean ± S.E. (*n* = 3–5). (See Table 2 for IC_50_s.) Inset shows example I_ACh_ traces from an oocyte expressing αβδε nAChRs before and following a 5-min. exposure to 1 μM α-EI; duration of each trace is 5 s. The block by α-EI is only slowly reversible.

To examine the mechanism by which αM-MIIIJ blocks the zebrafish nAChRs, we performed competition-blocking experiments between αM-MIIIJ and two other nAChR antagonists: α-BgTX and α-EI. I_ACh_ of all three subunit combinations were irreversibly blocked by α-BgTX (see Figure 9D–F) and slowly-reversibly blocked by α-EI (Figure 10 and its inset, also see Figure 9A–C).

When oocytes were exposed first to 10 μM αM-MIIIJ for 5-min., followed by a 5-min exposure to a mixture of 10 µM αM-MIIIJ and 1 μM α-EI, the block of I_ACh_ for all three subunit combinations of zebrafish nAChRs tested (αβγδ, αβδε, and αβδ) was rapidly reversed upon toxin washout (compare circles and squares in Figure 9A–C). The same was observed when α-BgTX (10 μg/mL) was used instead of α-EI with αβδ and αβδε nAChRs (Figure 9E,F), which indicates that these two nAChRs were largely protected by αM-MIIIJ against block by α-BgTX. However, for αβδγ nAChRs, αM-MIIIJ provided only partial protection against α-BgTX (Figure 9D).

### 2.8. αM-MIIIJ Blocks Indirectly-Evoked Mouse Muscle Action Potentials and Exogenously-Expressed Mouse and Human Muscle nAChRs

**Figure 11:**
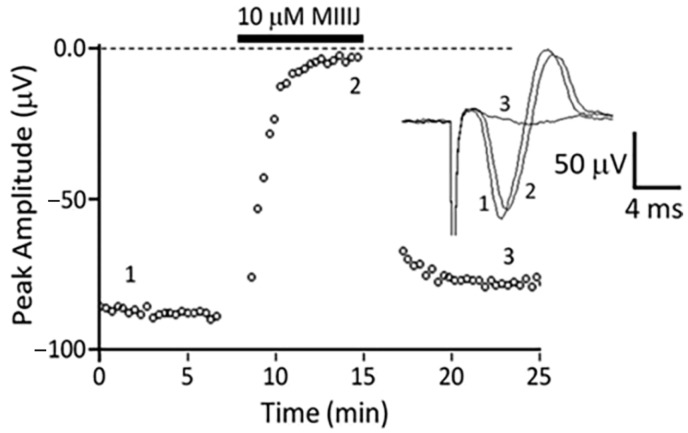
αM-MIIIJ (10 μM) blocks neuromuscular transmission in mouse muscle. Extracellular recording of the mouse levator auris longus (LAL) muscle’s action potential evoked by indirect stimulation was performed as described in Methods. Plot of peak (negative) amplitude of muscle action potential as a function of time before, during exposure to 10 μM αM-MIIIJ (indicated by black bar), and after toxin washout. Inset shows example traces of the extracellularly-recorded action potential before (trace 1), during block by toxin (trace 2) and after washout (trace 3), with trace numbers corresponding to times indicated in the main plot. The results are consistent with a low-potency block of mouse muscle nAChRs; direct evidence for this is illustrated in Figure 12.

**Figure 12:**
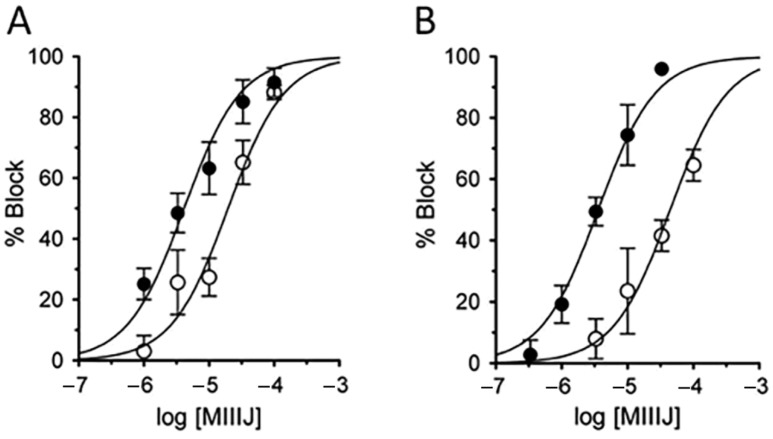
αM-MIIIJ blocks mammalian muscle nAChRs with low potencies. Oocytes expressing nAChRs from human (**A**) and mouse (**B**) were voltage-clamped and exposed to αM-MIIIJ as described in Methods. Concentration–response curves for the block by αM-MIIIJ of I_ACh_ in oocytes expressing αβδγ (open circles) or αβδε (closed circles) subunits. Solid lines represent best-fit curves to the Langmuir isotherm (see Table 2 for IC_50_s).

As described above, αM-MIIIJ failed to paralyze mice in in-vivo assays, and we wondered whether this was a consequence of inadequate dosage or possible pharmacokinetic effects. Thus, we tested the peptide on an isolated mouse muscle preparation and observed that 10 μM αM-MIIIJ blocked muscle action potentials evoked by motor-nerve stimulation (Figure 11). To more quantitatively assess the pharmacology of αM-MIIIJ, we tested the peptide on mammalian muscle nAChRs exogenously-expressed in *X. laevis* oocytes. At micromolar concentrations, αM-MIIIJ blocked I_ACh_ of αβδγ and αβδε nAChRs from both mouse and human (Figure 12). We conclude that αM-MIIIJ blocks both endogenous and exogenously-expressed mammalian muscle nAChRs, albeit with considerably less potency than muscle nAChRs of fish, the natural prey of *C. magus*.

### 2.9. Lack of Effects of αM-MIIIJ on Neuronal nAChRs

To examine αM-MIIIJ’s selectivity for muscle over neuronal nAChRs, we tested αM-MIIIJ on the following exogenously-expressed neuronal nAChRs: α3β4 (rat), α4β2 (rat), α9α10 (human). αM-MIIIJ (10 μM, the highest concentration tested) failed to block I_ACh_ of any of these receptors (Table 2).

### 2.10. Imaging the Binding of Fluorescently-Labeled α-BgTX at Frog NMJs

**Figure 13:**
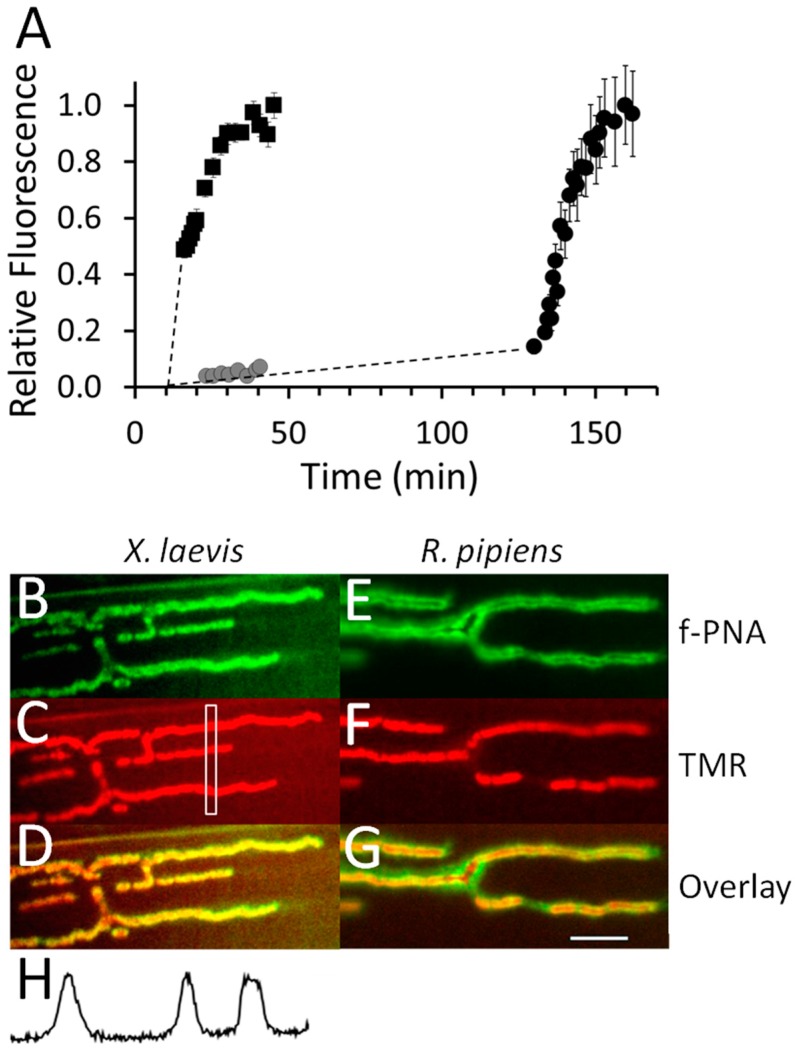
αM-MIIIJ (10 μM) inhibits binding of TMR α-BgTX at neuromuscular junctions of *X. laevis* LP muscle. Preparation of muscles, image acquisition, and quantification of fluorescence were performed as described in Methods. **A.** Two preparations, Muscle I and Muscle II, were stained with f-PNA so locations of NMJs could be identified as described in Methods. In Muscle I, the NFR bathing the muscle was replaced (at *t* = 10′) with 1 μg/mL TMR α-BgTX, and the NMJ was periodically imaged while its staining ensued until a steady state was achieved (squares). Muscle II was treated with 10 μM αM-MIIIJ (at *t* = 0) for 10-min. before it was exposed to 1 μg/mL TMR α-BgTX + 10 μM αM-MIIIJ, after which the endplate was periodically imaged for 30-min. (gray circles). Then the solution bathing muscle II was removed and reserved, and the muscle rinsed. After ~90-min., Muscle II was exposed to 1 μg/mL TMR α-BgTX alone while the NMJ was periodically imaged until the fluorescence reached steady state (black circles). The steady-state level of fluorescence varied from one NMJ to the next, so the fluorescence values at each endplate were normalized to the value obtained at steady state (at *t* ≈ 30′ for Muscle I and *t* ≈ 140′ for Muscle II). Each data point represents the mean ± SD from 9 to 12 regions of interest (ROIs; see rectangle in panel **C**) obtained along the NMJ. Presence of αM-MIIIJ clearly interfered with α-BgTX binding. **B** and **E**. f-PNA staining of an NMJ from *X. laevis* LP muscle (**B**) and *R. pipiens* CP muscle (**E**). **C** and **F**. TMR α-BgTX staining of *X. laevis* LP muscle (**C**) and *R. pipiens* CP muscle (**F**). Here, the CP muscle was treated with the reserved TMR α-BgTX + αM-MIIIJ solution removed from Muscle II; nevertheless, strong TMR staining is clearly evident. **D** and **G**. Overlays of images in panels **B** and **C** (**D**) and panels **E** and **F** (**G**). **H**. Fluorescence profile from the ROI in panel **C** (see rectangle therein). Calibration bar (20 μm, white line) in **G** applies to panels **B** through **G**. Presence of αM-MIIIJ clearly inhibited the binding of TMR α-BgTX at the NMJ of the *X. laevis* LP muscle but not at that of the *R. pipiens* CP muscle.

To further investigate αM-MIIIJ’s mechanism of action, we examined the manner in which the peptide affected α-BgTX binding. Tetramethylrhodamine conjugated α-bungarotoxin (TMR α-BgTX) readily stained NMJs of both *X. laevis* LP and *R. pipiens* CP muscles (Figure 13C,F, respectively). Muscles were pre-treated with fluorescein peanut agglutinin (f-PNA) so the progress of TMR α-BgTX binding at a given NMJ could be tracked (Figure 13B,E). In control experiments (i.e., in the absence of αM-MIIIJ), the binding of 1 μg/mL TMR α-BgTX to the *X. laevis* NMJ leveled off within about 20 min (Figure 13A, muscle I, squares). Similar results were obtained with *R. pipiens* CP preparations (cf., Figure 13F).

In contrast, when 10 μM αM-MIIIJ was present, very little binding of TMR α-BgTX to the *X. laevis* NMJ was observed even after 30 min (Figure 13A, muscle II, gray circles). Muscle II was washed for > 60 min. (to allow the αM-MIIIJ to unbind) and then exposed to TMR α-BgTX alone. This time, TMR α-BgTX readily bound and reached steady state in about 20 min (Figure 13A, muscle II, black circles), just as was observed for muscle I (Figure 13A, squares).

As a positive control, the ‘used’ mixture of α-BgTX + αM-MIIIJ to which muscle II had been exposed was applied to a ‘naïve’ *R. pipiens* CP preparation for about 10-min and imaged. The *R. pipiens* NMJs were robustly stained by TMR α-BgTX (Figure 13F), consistent with our results indicating that synaptic responses of *R. pipiens* CP muscles, unlike those of *X. laevis* LP muscles, are relatively resistant to block by αM-MIIIJ (Figure 3B). More to the point, these results also showed that: a) αM-MIIIJ had not somehow inactivated the α-BgTX to produce the protection represented by the gray circles in Figure 13A; and b) αM-MIIIJ’s association with nAChRs is reversible, consistent with electrophysiological results (e.g., Figure 2C, Figure 7 inset, Figure 8 and Figure 9).

### 2.11. Imaging the Binding of Fluorescently-Labeled α-BgTX at NMJs of Goldfish IC Muscle

We next examined whether αM-MIIIJ interfered with the binding of TMR α-BgTX to goldfish NMJs. We found that f-PNA could not be used as a marker for goldfish NMJs; so, goldfish IC muscle preparations were treated in two ways. Treatments involved exposure to TMR α-BgTX (1 μg/mL) for 10 min in the presence of αM-MIIIJ (10 μM, Treatment I) or in the absence of αM-MIIIJ (Treatment II). Preparations were then rinsed and imaged. Minimal TMR α-BgTX staining was observed in the presence of αM-MIIIJ (Figure S2A, Treatment I). On the other hand, robust staining was observed in the absence of the conotoxin (Figure S2A, Treatment II). These results demonstrate that, just as with *X. laevis* LP muscles, αM-MIIIJ protects goldfish IC muscles against α-BgTX binding.

### 2.12. Binding of Fluorescently-Labeled α-BgTX at Zebrafish NMJs

Goldfish intercostal muscle fibers have NMJs distributed over their entire surface (Figure S2B) and are therefore presumably fast muscles. To confirm our functional results, obtained with exogenously-expressed zebrafish fast and slow muscle nAChRs, on endogenous channels at the level of toxin binding, we exploited the fact that while NMJs of fast fibers are widely distributed, NMJs of slow fibers are largely confined to their myoseptal ends [30]. Toward this end, we performed competition-binding studies with fluorescently-labeled α-BgTX on a preparation from larval zebrafish, whose skin is relatively transparent and whose muscles have both fast and slow fibers, as described in Methods (see also Figure S3B).

This experiment employed Alexa488 α-BgTX (which fluoresces green) in addition to TMR α-BgTX, and it was performed in three phases. In phase 1, the preparation was lightly treated with TMR α-BgTX to provide an irreversible marker for NMJs. In the subsequent two phases, the preparation was treated with Alexa488 α-BgTX, first in the presence of αM-MIIIJ (phase 2), then in the absence of αM-MIIIJ (phase 3), with washing after each phase followed by imaging. About an hour elapsed between the end of phase 2 and the start of phase 3. The mean values of green (Alexa488 α-BgTX) fluorescence at NMJs of both fast and slow fibers were lower after Phase 2 than after Phase 3, showing that αM-MIIIJ (10 μM) inhibited α-BgTX binding to both types of muscle fibers (Figure S3A).

Taken together, the results of our electrophysiological and fluorescence-imaging experiments demonstrate that αM-MIIIJ avidly targets nAChRs at the NMJs of frog (*X. laevis*) and fish (*C. auratus* and *D. rerio*).

## 3. Discussion

### 3.1. αM-MIIIJ Uncovers a New Family of Muscle nAChR-Targeting Conotoxins

αM-MIIIJ and related sequences from other fish-hunters form a new class within the large M-superfamily of conotoxins. M-superfamily toxins are highly diverse and are found in nearly all cone snail species studied to date where they are known to exert various functions [21]. Two well-studied classes within the M-superfamily are μ-conotoxins which target voltage-gated Na channels [31] and ψ-conotoxins which block muscle nAChR without competing with the bungarotoxin binding site [20]. While these toxins all belong to the M-superfamily and share the same cysteine framework with αM-MIIIJ, their primary amino acid sequences are highly divergent (Figure 1A, bottom alignments). In contrast, αM-MIIIJ and related sequences from the *Pionoconus* clade are highly conserved and share a number of identical residues (Figure 1A, black arrowheads above top alignment). We demonstrate here that αM-MIIIJ blocks muscle nAChR and propose that the αM-MIIIJ-like peptides are also antagonists of muscle nAChR. Future functional studies are needed to investigate this hypothesis.

### 3.2. αM-MIIIJ Blocks Muscle nAChRs of X. laevis Frogs

Three ion-channel targets of known paralytic conotoxins are presynaptic V-gated Ca channels, postsynaptic nAChRs, and skeletal muscle V-gated Na channels (Table 1). With regard to *X. laevis* muscle, αM-MIIIJ did not block directly-evoked action potentials (Figure 2B), thus ruling out muscle Na channels as a target of the peptide. During exposure to αM-MIIIJ, no indication of quantized block (Figure 4A) or sparing of MEPSPs (Figure 4B) was observed, unlike the effects of ω-GVIA, which blocks presynaptic Ca channels at the frog NMJ [32]. Likewise, during the exposure to αM-MIIIJ, the block of EPSPs progressed smoothly with no major decrements (Figure 4A) that would suggest conduction failure in the motor axon; thus, we conclude that the peptide did not block action potentials (and by extension, V-gated Na channels) in the nerve. In contrast, αM-MIIIJ blocked the muscle’s response to ionotophoretically- (i.e., exogenously-) applied ACh (Figure 4C), clearly demonstrating that αM-MIIIJ is an antagonist of nAChRs.

### 3.3. αM-MIIIJ Blocks nAChRs of Goldfish Muscle

αM-MIIIJ is from a fish-hunting cone (see Introduction). Thus, the observation that the peptide paralyzes goldfish (Table S1) was not unexpected. With the knowledge that nAChRs were a target of αM-MIIIJ, we next focused our attention on nAChRs of goldfish muscle. αM-MIIIJ clearly blocked spontaneous miniature synaptic potentials in goldfish intercostal muscles (Figure 5).

### 3.4. αM-MIIIJ Blocks I_ACh_ of Zebrafish-Muscle nAChRs Exogenously Expressed in X. laevis Oocytes

The nAChR subunit compositions of fast and slow muscles of both larval and older zebrafish have been identified as αβδε and αβδ, respectively [25]. αM-MIIIJ blocked nAChRs composed of both subunit combinations (Figure 7). For good measure, we also tested the embryonic (αβδγ) combination and found it was also blocked by αM-MIIIJ (Figure 7). All three subunit combinations yielded αM-MIIIJ concentration–response curves with similar IC_50_s and slopes (Figure 7), with the αβδ combination having an affinity about a factor of two higher than that of the other two (Table 2). The αβε-subunit combination would be expected to have a subunit-stoichiometry for the pentameric nAChR of α_2_β_1_ε_2_, with two α/ε interfaces, while the αβδε-combination would have one α/ε interface (and one α/δ interface). The IC_50_ of αM-MIIIJ for αβε was similar to that for αβδ (Table 2). It might be noted that for a receptor with two binding sites with identical affinity for a toxin, the IC_50_ for functional block of the receptor would be the square-root of the affinity constant of the sites [33]. These results suggest that αM-MIIIJ binds with about equal avidity to both the α/δ and α/ε interfaces, which is consistent with our αBgTX-competition experiments (Figure 9E,F). αM-MIIIJ provided αβδγ nAChRs only-partial protection against α-BgTX (Figure 9D), suggesting that αM-MIIIJ had a lower affinity for the α/γ-interface than the α/δ- and α/ε-interfaces; alternatively, α-BgTX bound to the α/γ interface more quickly than to either the α/δ- or α/ε-interfaces. This issue may be resolved when functional expression of αβγ nAChRs is achieved.

### 3.5. αM-MIIIJ Inhibits the Binding of α-BgTX to Frog and Fish NMJs

Fluorescently-tagged α-BgTX has long served as an extremely useful marker for nAChRs since the initial synthesis of TMR α-BgTX and its use to stain *R. pipiens* and *X. laevis* NMJs [34]. We observed that αM-MIIIJ inhibited the binding of α-BgTX at the NMJs of *X. laevis* (Figure 13A), goldfish (Figure S2A) and zebrafish (Figure S3A). As might be expected, αM-MIIIJ did not interfere with the binding of α-BgTX at the NMJ of *R. pipiens* (Figure 13F).

It is intriguing that αM-MIIIJ blocks muscle nAChRs of *X. laevis* but not those of *R. pipiens*. This result is consistent with our observations that αM-MIIIJ protects nAChRs of *X. laevis* (Figure 13A), but not those of *R. pipiens* (Figure 13F), against α-BgTX. It would be interesting to explore the underpinnings of the difference at the molecular level. It might be noted that neuronal nAChRs of closely-related frogs can have different sequences, as was observed where the driving force for variation involved resistance to epibatidine, a nAChR-targeting neurotoxic alkaloid used by some frogs for chemical defense [35].

### 3.6. Possible Rationale for the Presence of both a-MI and αM-MIIIJ in C. magus Venom

Aside from α-MI and αM-MIIIJ, each of the *C. magus* venom peptides listed in Table 1 has a distinct ion-channel target. α-MII was much more potent than α-MI in blocking synaptic transmission in sympathetic ganglia of frog, which led us to speculate that the role of α-MII in *C. magus* venom is to interfere with the flight-or-fight response of fish prey [36], in contrast to the role of α-MI, which is to immobilize fish prey.

α-MI is potent in paralyzing mice, and in view of its sequence homology to α-GI, α-GIA and α-GII (Table 3), which block muscle nAChRs [37], α-MI was presumed to also block muscle nAChRs [8]. Indeed, competition-binding experiments with [^125^I]α-BgTX revealed that α-MI blocks muscle nAChRs by binding with high affinity to the α/γ (of Torpedo) and α/δ (of mouse) ACh-binding sites of the receptor [22,38]. α-MIC, the most recently characterized α-MI congener, has a similar LD_50_ against fish (*Xiphophorous helleri*) as that of α-MI; furthermore, α-MIC competitively inhibits the binding of [^3^H]epibatidine to the muscle subtype of nAChRs from *Torpedo californica* with a K_i_ similar to that of α-MI [39].

The disparity between the structures of α-MI and αM-MIIIJ raises the question as to whether αM-MIIIJ binds to the same site of muscle nAChRs as does α-MI. Our results showing that 10 μM αM-MIIIJ almost completely inhibits α-BgTX binding (Figure 13A, Figures S2A and S3A) suggest that at this concentration αM-MIIIJ likely occludes both α-BgTX-binding sites of a given nAChR. These results are consistent with the protection αM-MIIIJ affords the zebrafish nAChR subunit combinations αβδ (slow-fiber nAChRs) and αβδε (‘adult’ fast fiber) against block of their I_ACh_ by α-BgTX (Figure 9E,F). The only-partial protection 10 μM αM-MIIIJ afforded the αβδγ nAChR subunit combination (Figure 9D) suggests that α-BgTX may bind more quickly to the α/γ interface than to either the α/δ or α/ε interfaces. Alternatively, αM-MIIIJ may have a lower affinity for the α/γ interface than for either the α/δ or α/ε interfaces. We did not perform any experiments on embryonic zebrafish muscle, which express αβδγ nAChRs. In view of our electrophysiological results of Figure 9D, we predict that αM-MIIIJ would only partially protect NMJs of embryonic muscle against staining by fluorescent α-BgTX.

In the discussion above, we assumed that αM-MIIIJ interacts directly with each of the two ACh-binding (or orthosteric) sites of the receptor. A far less parsimonious possibility is that αM-MIIIJ modulates the ACh-binding sites of the receptor by binding at an allosteric site. The rationale for entertaining the latter possibility is to understand why *C. magus* venom has two structurally divergent peptides (αMI and αM-MIIIJ) to accomplish the same physiological endpoint—incapacitation of the nAChR. That is, perhaps α-MI acts orthosterically while αM-MIIIJ acts allosterically. This issue may be resolved by experiments involving mutagenesis of specific residues in the nAChR subunits or by examining whether αMI and αM-MIIIJ can act synergistically.

A species of fish-hunting *Conus* with three subfamilies of peptides that target muscle nAChRs is *C. ermineus*, whose venom contains α-EI (an α-4/7 subfamily member), αA-EIVA/B (two closely-related αA-subfamily peptides that differ in only two residues), and the more recently-discovered α-EIIA (Table 3). α-EI binds preferentially to the αδ interface of mouse nAChRs whereas αA-EIVA binds equally well to both the αδ and αγ interfaces [33]. Our tests with α-EI on zebrafish muscle nAChRs indicate that it blocks all three subunit combinations (αβδ, αβδε, and αβδγ) with similar potencies (Figure 10). Furthermore, αM-MIIIJ completely protected against the block by α-EI of all three subunit combinations (Figure 9A–C). It remains to be seen which sites αA-EIVA binds to on zebrafish muscle nAChRs.

*C. purpurascens* is a fish-hunter with nAChR-targeting conotoxins belonging to three subfamilies: α-PIB, αA-PIVA, and ψ-PIIIE/F (Table 3). ψ-PIIIE blocks I_ACh_ of *X. laevis* oocytes expressing *Torpedo* nAChRs but does not compete with [^125^I]α-BgTX binding to *Torpedo* nAChRs [20]. Although the binding site of ψ-PIIIE remains to be identified, it is clear that *C. purpurascens* venom has subfamilies of conotoxins that block nAChRs via different modes or mechanisms. In this vein, it might be noted that *C. imperialis*, a worm-hunting species of *Conus*, has two closely related α-conotoxins that block α7 nAChRs, α-ImI and α-ImII [58,59]. While α-ImI competes with α-BgTX, α-ImII does not [58]. The binding of α-ImII to α7 nAChRs has been investigated [60], and its binding site and mechanism of action were given the moniker “monkey wrench” [61].

It might be noted that there is the possibility that αM-MIIIJ may act promiscuously, and its more (or equally) important target in the wild is an ion channel other than muscle nAChRs; only further testing will resolve this issue. There is precedent for a conotoxin affecting the function of more than a single ion-channel target, for examples: μO-conotoxin MrVIA/B can block both V-gated Na and V-gated Ca channels of snail neurons [62,63] (in addition to blocking vertebrate V-gated Na channels [64,65,66]); μ-conotoxin CnIIIA, originally shown to block V-gated Na channels [12] can also block neuronal nAChRs [67]; μ-conotoxins PIIIA and SIIIA, originally shown to block Na channels [15,23] can also block K channels in the K_V_1 family [68]. Recently, an α-conotoxin from *C. eburneus*, Eu1.6, was characterized that blocked (when tested at 1 μM) N-type voltage-gated Ca channels in dissociated mouse dorsal root ganglion neurons. Among various rat nAChRs exogenously expressed in *X. laevis* oocytes, 10 μM Eu1.6 blocked α7 nAChRs best (where I_ACh_ was attenuated by about a half) but had no effect on skeletal muscle nAChRs with a subunit composition of αβδε [69].

## 4. Conclusions

αM-MIIIJ blocks the function of nAChRs in the postsynaptic membrane of skeletal muscles of fish and frogs. The peptide also interferes with α-BgTX binding to these receptors. It is well established that α-BgTX blocks nAChR function by occluding the ACh-binding sites on the receptor; by extension, we conclude that αM-MIIIJ blocks nAChR function by interfering with ACh binding to nAChRs, although it remains to be seen by how similar a mechanism as that of α-BgTX.

We reveal that αM-MIIIJ belongs to a new class of toxins from the M-superfamily and identify several toxin sequences with significant sequence homology to αM-MIIIJ. We hypothesize that these and additional homologs likely to be identified in the future will also function as muscle nAChR antagonists. These studies will expand our understanding of the biology of fish hunting and the versatility of toxins belonging to the M-superfamily, and further highlight the remarkable adaptability of toxin genes in these marine predators.

## 5. Materials and Methods

### 5.1. αM-MIIIJ Purification and Sequencing

Crude venom was extracted from *C. magus* (~200 specimens) collected in waters surrounding the island of Marinduque in the Philippines. Lyophilized venom was extracted with 1.1% v/v acetic acid and chromatographed on a column of Sephadex G25 (2.5 × 92 cm) as previously described (McIntosh et al., 1982 [8], method 3). Low molecular mass fractions were subsequently separated by reversed-phased liquid chromatography (HPLC) on a Supelco LC18 semi-prep column (10 mm id × 25 cm; 5 μm particle size) at a flow rate of 3.0 mL/min. A 0.1% TFA–0.01% TFA, 60% acetonitrile buffer system was used for elution. The gradient was 15% B × 5 min, increasing to 25% B at 25 min and increasing to 60% B at 35 min. Absorbance was monitored at 210 nm.

Peptide from the final purification was stored in the HPLC buffer in which it eluted. A 287-μL solution of this purified peptide (~250 pmol) was combined with 14.4 μL (20:1 v/v) of 0.5 M Tris base which raised the pH to a value between 7 and 8 as measured with pH paper. Seventy-five μL of 50 mM dithiothreitol was added (final concentration 10 mM); the reaction vessel was flushed with argon, and the reaction incubated at 65 °C for 15 min. The solution was allowed to cool; 15 μL of 20% 4-vinyl pyridine in ethanol was added, and the solution was reacted for a further 25 min at room temperature in the dark. The solution was diluted 3-fold with 0.1% trifluoroacetic acid, and the alkylated peptide was purified on the Brownlee column.

Sequencing was performed with Edman chemistry on an Applied Biosystems 477A (Applied Biosystems, Foster City, CA, USA) Protein Sequencer at the Protein/DNA Core Facility at the University of Utah Cancer Center (Salt Lake City, UT, USA). Mass spectrometry was performed as described previously [9].

Initial attempts at sequencing yielded no sequence suggesting that the N-terminal residue might be pyroglutamate as found in previously characterized conotoxins. We therefore subjected the peptide to digestion with pyroglutamyl aminopepdtidease in an attempt to remove the potential pyroglutamate and allow sequencing.

An amount of 400 μg of calf liver pyroglutamyl aminopepdtidease (Boehringer) enzyme powder (~16 µg protein) and ~1 nmol of αM-MIIIJ was dissolved in 100 µL of incubation buffer consisting of 50 mM Na_2_HPO_4_, pH 7.3, 10 mMβ-mercaptoethanol, 1 mM EDTA and incubated at 37 °C for 30 min. The reaction products were then purified by HPLC using a Vydac C18 4.6 mm ID column using buffer A = 0.1% TFA and buffer B = 0.1% TFA, 60% acetonitrile buffer. The gradient was 20% buffer B increasing to 50% buffer B over 30 min The resulting product was successfully sequenced.

### 5.2. Identification of Homologous Sequences

Using the mature toxin sequence of αM-MIIIJ we searched for homologous sequences in *C. magus* and additional taser-and-tether hunting species from the *Pionoconus* clade (*Conus consors* and *Conus striatus*) in published venom gland transcriptomes [14,17,70] using the Basic Local Alignment Search Tool (BLAST) [71] with an e-value of <1 × 10^−1^.

### 5.3. αM-MIIIJ Synthesis

The peptide was manually assembled by solid-phase approach [72]. The starting Boc-Cys(Mob)-CM resin with a capacity of 0.3 mmol/g was obtained according to published procedures [73]. All Nα-tert-butyloxycarbonyl (BOC) protected amino acids with side chain protection were purchased from Bachem Inc. (Torrance, CA, USA). The side chain protecting groups were as follows: Arg(Tos), Asp(OChx), Cys(Mob), Lys(e-2ClZ), Ser(Bzl), Tyr(2BrZ). Z in the sequence represents pyroglutamic acid.

Three equivalents of Boc-amino acids based on the original substitution of the resin were used for each coupling. The couplings of the protected amino acids were achieved by adding three equivalents coupling reagents BOP (Benzotriazole-1-yl-oxy-tris(dimethylamino)-phosphoniumhexafluoro- phosphate) or HBTU (*O*-(benzotriazol-1-yl)-*N*,*N*,*N*′,*N*′-tetramethyluronium hexafluorophosphate) in dichloromethane and dimethylformamide, respectively to the resin and adjusting the pH to 9 with diisopropylethylamine (DIPEA) then shaking it for 20 min. Completions of the couplings were monitored by the qualitative ninhydrin test [74]. Boc removal was achieved by treatment with 60% trifluoroacetic acid and 3% ethanedithiol in CH_2_Cl_2_ for 30 min. An isopropyl alcohol (containing 1% ethanedithiol) wash followed the TFA treatment and then successive washes with triethylamine solution (10% in CH_2_Cl_2_), methanol, triethylamine solution, methanol and CH_2_Cl_2_ completed the neutralization. The completed peptide was cleaved from the resin support with simultaneous side chain deprotection by using anhydrous hydrogen fluoride containing the scavengers anisole (10% v/v) and methyl sulfide (1% v/v) at 0 °C for 75 min. The diethyl ether-precipitated crude peptide was extracted from the resin with 50% isopropanol/ 50% water solution, the pH was adjusted to 8–8.5 with DIPEA and the mixture was slowly stirred for several days to complete the cyclization that was monitored by the Ellman test [75]. The peptide was purified by preparative HPLC. The cartridge used was hand packed, in house, with Waters polyethylene sleeve and frits and reversed-phase 300 Å Vydac C_18_ silica (15–20 µm particle size). The peptide was eluted with the solvent system 0.1% TFA/H_2_O/CH_3_CN at a flow rate of 100 mL/min. A linear gradient 1.5% B per 1 min increases from the baseline %B (Eluent A = 0.1% TFA/H_2_O, eluent B = 60% CH_3_CN, 40% A) was applied. Capillary zone electrophoresis (CZE) was carried out on a Beckman P/ACE System 2050 controlled by an IBM Personal System/2 Model 50Z connected to a ChromJet integrator. CZE analysis employed a field strength of 10–20 kV at 30 °C with a buffer of 15% CH_3_CN /85% 100 mM sodium phosphate pH 2.5 on a Beckman eCAP capillary (363 mm o.d. 75 mm i.d. 50 cm length), LSIMS mass spectra were obtained with a JEOL JMS-HX110 double-focusing mass spectrometer (JEOL, Tokyo, Japan) fitted with a Cs+ gun.

The experiments described in this report employed synthetic αM-MIIIJ.

### 5.4. Animal Use

Use of all animals in this study followed protocols approved by the University of Utah Institutional Animal Care and Use Committee (IACUC) that conform to the National Institutes of Health Guide for the Care and Use of Laboratory Animals. IACUC protocols were as follows: #14-08018 (approved period, 20 August 2014–19 August 2017) and #17-07020 (approved period, 01 August 2017–31 July 2020). Mice were euthanized with CO_2_. *X. leavis* and fish were anesthetized with Tricaine (0.4% for *X. laevis* and goldfish, 0.02% for zebrafish) and decapitated. *R. pipiens* were sacrificed by double pithing.

### 5.5. In Vivo Bioassays of αM-MIIIJ on Goldfish and Mice

*Carassius auratus* (goldfish). Toxin was dissolved in 0.9% w/v NaCl (NSS), and 10 μL were injected into the dorsal epaxial muscle of a given fish. The fish was then placed in a beaker (14.5 cm dia. × 7.5 cm height) containing ~700 mL of water with a 35-mm long, 10-mm diameter magnetic stir bar rotating at ~200 RPM creating a vortex, such that the fish must actively swim to avoid being trapped in the center. Each of three control fish injected with NSS alone were able to swim with no sign of being overpowered by the vortex for >35 min. Fish injected with αM-MIIIJ gradually became paralyzed within several minutes and were unable to escape the vortex. After paralysis, fish were moved to 250 mL beakers to be observed. Motion of the mouth and gills was observed, but fish were unable to swim and did not respond to prodding of fins, dorsal and ventral sides, and eyes.

Mice. Swiss Webster mice (~13 g avg. weight) were injected intraperitoneally (i.p.) with ~2.3 nmol αM-MIIIJ/gram mouse dissolved in 30 μL NSS. Control mice were injected with 30 μL NSS only. After injection, mice were observed for >1.5 h for signs of paralysis.

### 5.6. Isolated Muscle Preparations

*Rana pipiens* (grass frog). The cutaneous pectoralis (aka cutaneus pectoris, CP) muscle of *Rana pipiens* (~65 mm) long adults of both sexes) was used. This flat, thin muscle has long served as a go-to preparation for studies of the structure and function of frog neuromuscular synapses (see e.g., [34,76,77]). For extracellular recording, the muscle was prepared and used as previously described [78]. For fluorescence microscopy, the preparation was pinned with 0.1 or 0.2 mm dia. stainless steel Minutien pins to coverslips (22 mm dia.) coated with Sylgard (Dow Corning, Midland, MI, USA), a transparent silicone elastomer.

*Xenopus laevis* (African clawed frog). These frogs do not have CP muscles, therefore we used their longitudinal pectoralis (LP) muscles, which are flat muscles that lie laterally on either side of the rectus abdominus muscle of the animal [79]. The motor nerve contacts the muscle near the rostral end and sends branches that run parallel with the muscle fibers. For extracellular recording, strips of LP muscles of adult animals were used. A given strip was placed in a multi-well chamber fabricated from Sylgard where the wells, each 4-mm diameter and 4-mm deep, were in a linear array with a 1 mm-partition separating one well from the next. The rostral end of the strip was placed in well 1 and the caudal end in well 5 to 8, depending on the strip’s length. The portion of the muscle strip overlying a partition was covered with Vaseline. A pair of stimulating electrodes were located in wells 1 and 2 or wells 2 and 3; with a ground electrode located in well 3 in the former case, and in well 4 in the latter. A pair of recording electrodes were located in adjacent wells 4 and 5, 5 and 6, 6 and 7, or 7 and 8 (depending on muscle length). To obtain indirectly-evoked muscle action potentials without confounding, directly-evoked muscle action potentials, 10 μM μ-conotoxin PIIIA, which specifically blocks the muscle subtype of V-gated Na channels [23], was present in the two wells in which the stimulating electrodes were located; thus, only the motor nerve was directly activated by the electrical stimulus. In a separate set of experiments, muscle action potentials in response to direct stimulation were recorded by having 10 μM d-tubocurare in all wells to block synaptically-mediated responses. Excitatory postsynaptic responses evoked by indirect stimulation were recorded by placing 10 μM μ-conotoxin PIIIA in all wells.

For intracellular recording and fluorescence microscopy, we used LP muscles from ~5-cm juveniles of either sex. Cutting the muscle longitudinally approximately in half, a procedure that did not denervate the majority of the fibers in the medial half, allowed the remaining muscle to fit in a ~23-mm diameter, ~2-mm deep recording chamber or be pinned onto a Sylgard-coated coverslip (22 mm dia.).

*Carassius auratus* (goldfish). We discovered that the intercostal (IC) muscles of adult goldfish can be easily prepared for electrophysiology and are readily amenable for fluorescence microscopy following vital staining. Briefly, goldfish (~4-cm long) of undetermined sex were anesthetized with 0.4% Tricaine, decapitated, and its skin descaled by plucking with serrated forceps. A pair of sharp-tipped scissors was used to make a longitudinal incision in the body wall along the ventral midline from anal fin to pelvic fin, with care taken to avoid cutting any entrails. This was followed, on each side of the animal, by two vertical incisions in the body wall from the ventral midline, dorsally to the spine: one from the anal fin and the other from the pelvic fin. Finally, each half of a rib cage, with its skin intact, was cut along the spine to free each hemi-rib cage from the rest of the animal. Each hemi-rib cage was pinned, skin-side down, in a Sylgard-coated plastic culture dish containing NFR, and the iridophore-laden parietal peritoneal integument overlying the inner surface of the ribs was carefully stripped away manually with tweezers to expose the two diagonally-overlapping layers of intercostal muscles. The intercostal muscle preparation, consisting of at least four ribs, was then pinned skin-side down to a Sylgard-coated coverslip (22-mm diameter). Two such preparations can be readily obtained from each side of a given goldfish.

*Danio rerio* (zebrafish). Briefly, larval zebrafish (4- to 6-day old), anesthetized with 0.02% Tricaine and decapitated, were pinned on their sides to a Sylgard-coated coverslip (15-mm dia.) with electrolytically-sharpened tungsten pins [80] essentially as described by Brehm’s laboratory [81]. The skin on the uppermost side of each fish was then peeled away so toxins could readily access the underlying body musculature [81,82]. The transparency of the skin in these larval fish allowed the muscle fibers closest to the coverslip to be readily imaged with an inverted microscope. Slow fibers are distinguished by nAChRs localized largely to the myoseptal regions (at the ends of the muscle fibers), while fast muscles are invested with nAChRs distributed in clusters or puncta dispersed throughout a given muscle fiber (for review see [30]).

Mice. The levator auris longus (LAL) muscle initially described for electrophysiology by Faille and coworkers was used [83]. This thin, flat muscle preparation, which allowed ready access and egress of drugs, was pinned in a rectangular trough (~5 × 15 × 4 mm deep) and the motor nerve was draped across two adjacent wells (~4 mm dia. × 4 mm deep) and into the muscle-containing trough (all partitions were 1 mm wide and they, along with the overlying nerve, were covered with Vaseline). The chamber was fabricated from Sylgard.

Solutions. Both frog and fish muscle preparations were bathed in normal frog Ringer’s (NFR), which consisted of (in mM): NaCl (111); KCl (2); CaCl_2_ (1.8); and half-Na HEPES (10 mM), pH 7.2. Mouse muscle preparations were bathed in normal mammalian Ringer’s (NMR) composed of (in mM): NaCl (145); KCl (5); CaCl_2_ (2); sodium citrate (1); glucose (10); and half-Na HEPES (10 mM), pH 7.4.

### 5.7. Muscle Electrophysiology

Extracellular recordings were acquired with a pair of wire electrodes connected to a Grass P55 A.C. preamplifier at an amplification of 100 or 1000 and bandpass filtered with −6 dB cut-off frequencies of 1 Hz (unless otherwise indicated) and 1 kHz. The output of the preamp was digitized at a sampling rate of 10 kHz with an NI PCI-6221 data acquisition board (National Instruments, Austin, TX, USA). Motor-nerve stimulation was accomplished with either a pair of wire electrodes, one each in the nerve-containing wells (*R. pipiens* CP, adult *X. laevis* LP, mouse LAL preparations) or a suction electrode (juvenile *X. laevis* LP preparation). The stimulating electrodes were connected to a Grass Stimulus Isolation Unit (“SIU5A”) driven by a Grass S88 stimulator (Grass Instruments, Quincy, MA, USA) that was set to deliver a supermaximal stimulus (~10 V, 0.1-ms rectangular pulse) at a frequency of 1/20 sec. Responses were recorded with a pair of extracellular recording electrodes in the trough: one electrode located near the middle of the muscle, and the other near the caudal end of the muscle. A ground electrode was located in the trough between the stimulating and recording electrodes. All extracellular electrodes were stainless steel. For all electrophysiological recordings, timing of stimuli, and data acquisition, storage and retrieval were performed with home-made software written with LabVIEW (National Instruments, Austin, TX, USA).

Intracellular recordings were acquired with glass microelectrodes filled with 3 M KCl (*X. laevis* LP muscle) or 4M K-acetate (goldfish IC muscle) (~5 MΩ resistance) and a MultiClamp 700B amplifier (Molecular Devices, Sunnyvale, CA, USA) in current-clamp mode with current clamped at zero pA, where membrane potentials were amplified 10-fold, high-frequency filtered at 1 kHz. (Note, KCl is the conventional electrolyte used to fill intracellular microelectrodes for recording from frog skeletal muscle (e.g., ref. [84]). Although K-acetate-filled microlectrodes are less popular, they have been used to record from goldfish skeletal muscle (see e.g., [85] We used 3 M KCl and 4M K-acetate solutions because we happened to have these on hand.) The output of the amplifier was digitized and synchronized with the stimulator using the hardware and software described above for extracellular recording. Insertion of the intracellular electrode at a location near an NMJ of an *X. laevis* LP fiber was assisted by use of fluorescein peanut agglutinin (f-PNA, see Section 5.9 below). NMJs of goldfish IC fibers are distributed all along a given fiber, so spontaneous miniature excitatory potentials (MEPSPs) were readily recorded regardless of electrode placement. Amplitudes of MEPSPs were measured manually.

Acetylcholine (ACh) iontophoresis was performed in a manner similar to that previously described [84]. In essence, a Grass Instruments S-44 stimulator in conjunction with a home-made stimulus isolation unit (SIU) was used. One output of the SIU led to one terminal of “brake box” consisting of a 9-V battery whose output was regulated by a potentiometer. The other terminal of the brake box was connected to a 100 MΩ current-limiting resistor located close to, and in series with, a chlorided silver wire that was inserted into a glass micropipette filled with 1 M ACh•Cl and whose resistance was about 10 MΩ. The other output of the SIU let to a chlorided silver wire bath electrode that provided the return path for ACh current. A (negative) braking current of ~15 nA was used to prevent desensitization-inducing ACh from leaking out of the pipette, while ACh was ejected from the micropipette with a 10-ms (positive) current pulse whose amplitude was adjusted to obtain ACh-gated depolarizations in the ~10 mV range. The ACh-filled micropipette was placed near an NMJ under visual control assisted by the use of f-PNA (see Section 5.9 below).

### 5.8. Voltage-Clamp of X. laevis Oocytes Exogenously Expressing Muscle nAChRs from Zebrafish, Mouse, and Human

cDNA clones of α-, β- (β1b isoform), γ-, ε-, and δ-subunits of the muscle subtypes of nAChR from zebrafish [25] were generously provided by Paul Brehm. cRNA were produced from these clones by linearizing with BamHI and transcription with T7 (mMessage mMachine T7 kit, Invitrogen, Carlsberg, CA, USA). cRNA injections and voltage-clamping of ACh-gated currents were done essentially as previously described [9]. Briefly, a given oocyte was injected with ~1 ng of cRNA for each nAChR subunit in the following combinations: αβδγ (fast embryonic muscle) or αβδε (fast larvae and adult muscle) or αβδ (slow muscle) and incubated at 16 °C for at least one day. The same was done for the αβε subunit combination except ~10 ng of cRNA were injected for each subunit. Preparation and injection into oocytes of the following materials were as previously described: cDNA for mouse and human muscle nAChR [55]; cRNA for rat α3β4 and α4β2 and human α9α10 nAChRs [9,86].

To record ACh-gated currents (I_ACh_), an oocyte was placed in a 30 μL chamber, perfused with ND96 (96.0 mM NaCl, 2.0 mM KCl, 1.8 mM CaCl2, 1.0 mM MgCl2, 5 mM Hepes, pH 7.1–7.5) containing 0.1 mg/mL bovine serum albumin, two-electrode voltage clamped at a holding potential of −70 mV with an OC725C Oocyte Clamp amplifier (Warner Instruments, Hamden, CT, USA), and exposed to a 1-s pulse of ACh (10 μM for all nAChRs except zebrafish αβε-subunit combination where [ACh] was 100 µM) delivered at a frequency of 1/min. Oocytes were exposed to toxin in a static bath for 5-min. by halting the perfusion (and ACh pulses) and applying 3 μL of toxin to the chamber at 10-times the final concentration and stirring the bath by aspirating and expelling the mixture with a pippetor. The same was done for control responses except 3 μL ND96 was introduced into the static bath instead of peptide. After the five minutes had expired, the ACh pulses (and perfusion) were resumed. The peak amplitude of the first I_ACh_ response after the 5-min ND96 control exposure served as the control response, and the peak amplitude of the first I_ACh_ response after the 5-min toxin exposure served as the test response. The ratio of the test/control response was multiplied by 100 to obtain the percentage response. Each data point of a concentration–response curve represents the average ± S.E. of at least three oocytes. Concentration–response curves were fit, with Prism software (GraphPad Software Inc., San Diego, CA), to the Langmuir adsorption isotherm: % Block = 100%/{1 + (IC_50_/[toxin])}.

### 5.9. Fluorescence Imaging

#### 5.9.1. Imaging of Fluorescently-Tagged α-bungarotoxin (α-BgTX) and Fluorescein Peanut Agglutinin (f-PNA) Bound at NMJs

Tetramethylrhodamine (TMR) α-BgTX and fluorescein PNA were used essentially as first described by the laboratories of Cohen and Ko, respectively [34,87]. TMR α-BgTX and Alexa-488 α-BgTX were purchased from Molecular Probes (Eugene, OR, USA) and used at concentrations and exposure times detailed in Results. Fluorescein-PNA was purchased from Vector Laboratories (Burlingame, CA, USA), and muscles were exposed to it at a concentration of 50 μg/mL for 20–30 min. f-PNA was originally used with *R. pipiens* NMJs, where it was shown not to adversely affect synaptic transmission [87]. We found that f-PNA also could serve in that capacity with *X. laevis* LP muscles (see Figure 13B); in contrast, we found that f-PNA was not useful as a marker for NMJs in fish.

Frog and goldfish muscle preparations were imaged with a Nikon FN-1 (upright) microscope fitted with a Nikon Apo 40X/0.8 water-immersion objective and a Nikon Digital Sight DS-Qi1Mc camera using a 2-s exposure at a gain of 8. Zebrafish muscle preparations were imaged with a Nikon Ti (inverted) microscope fitted with Nikon Plan Fluor 40X/0.75 objective and a Retiga SRV-1394 camera (QImaging, Surrey, BC, Canada) with a 1-s exposure at a gain of 10. All images were acquired and processed with Nikon NIS-Elements software.

#### 5.9.2. Fluorescence Intensity Measurements

The level of fluorescently tagged α-BgTX bound at NMJs was quantified as follows. For frog muscles, a rectangular region of interest (ROI) with a width of 16 pixels (2.56 μm) was placed over the NMJ with the ROI’s long axis perpendicular to the long axis of the NMJ. A profile plot was obtained from the ROI where the plot’s Y-axis represented fluorescence intensity averaged over the pixels in the short dimension of the ROI, and the plot’s X-axis represented distance (in μm) along the long dimension of the ROI (for an example, see Panels C and H in Figure 13). The value of the profile’s peak minus that of the flanking baseline was taken as the fluorescence-intensity level. The same was done for fish muscles except the width of the rectangular ROI was narrower (6 pixels), and only NMJs were measured whose larger dimension exceeded the width of the ROI (see Figure S2B).

### 5.10. Toxins and Their Application

αM-MIIIJ was synthesized as described in Results. α-conotoxin EI (α-EI), from *C. ermineus*, was synthesized as previously reported [22]. Toxins were dissolved in distilled water, NFR, NMR, or ND96, and stock solutions were kept frozen until use. Unless indicated otherwise, the solution bathing the preparation was manually removed and replaced by a toxin solution; static baths were used throughout to conserve toxin. Toxins were washed out by manually replacing the bath solution with NFR or NMR; in the case of oocytes, the bath was gravity perfused with ND96.

All experiments were performed at room temperature.

## Figures and Tables

**Table 1:** Sequences and targets of paralytic conotoxins from *C. magus*.

Peptide	Sequence ^1^	Ion-Channel Target	Reference
α-MI	GR**CC**HPA**C**GKNYS**C**#	muscle nAChR	McIntosh 1982 [8]
α-MII ^2^	G**CC**SNPV**C**HLEHSNL**C**#	neuronal nAChR	Cartier 1996 [9]
ω-MVIIA	**C**KGKGAK**C**SRLMYD**CC**TGSCRSGK**C**#	presynaptic Ca channel	Olivera 1987 [10]
μ-MIIIA	ZG**CC**NVPNG**C**SGRW**C**RDHAQ**CC**#	muscle Na channel	Wilson 2011 [11] Zhang 2006 [12]
αM-MIIIJ	ZK**CC**SGGS**C**PLYFRDRLI**C**P**CC**	muscle nAChR	Rybin 2020 [this report]

^1^ Cysteine residues are bolded to highlight them; # indicates C-terminal amidation; Z indicates pyroglutamate residue. ^2^ α-MII is not paralytic but is shown here for comparison with α-MI.

**Table 2:** IC_50_s of αM-MIIIJ and α-EI on nAChRs expressed in *X. laevis* oocytes. ^1^.

nAChR ^2^	αM-MIIIJ	α-EI
zαβδ	0.033 (0.023–0.048) ^3^	0.073 (0.045–0.118) ^4^
zαβδγ	0.061 (0.049–0.076) ^3^	0.103 (0.069–0.155) ^4^
zαβδε	0.058 (0.040–0.085) ^3^	0.058 (0.039–0.087) ^4^
zαβε	0.034 (0.022–0.053) ^5^	N. A.
mαβδγ	44.4 (25.4–77.5) ^6^	N.A.
mαβδε	3.6 (2.6–4.9) ^6^	N. A.
hαβδγ	19.6 (13.8–27.7) ^6^	N. A. ^7^
hαβδε	4.1 (2.9–5.9) ^6^	N. A.
hα9α10	>>10 ^8^	N. A.
rα4β2	>>10 ^8^	N. A. ^9^
rα3β4	>>10 ^8^	N. A. ^10^

^1^ IC_50_ value in μM, 95% C.I. in parentheses. N.A. indicates not tested or not available (but see related information in enumerated footnote). ^2^ Prefix: z, zebrafish; m, mouse; h, human; r, rat. Greek letters: combination of nAChR-subunit cRNAs injected into a given oocyte. ^3^ from Figure 7. ^4^ see Figure 9. ^5^ see Figure 8A. ^6^ see Figure 11. ^7^ IC_50_ = 0.187 ± 0.043 μM (mean ± S.E.) from patch-clamped human rhabdomyocarcoma cell line TE671 expressing hαβγδ [28]. ^8^ No block observed with 10 μM, the highest αM-MIIIJ concentration tested (*n* = 3 oocytes). ^9^ I_ACh_ in patch-clamped HEK293 line expressing hα4β2 nAChRs was 60% blocked by 10 μM α-EI [28]. ^10^ I_ACh_ in patch-clamped HEK293 line expressing hα3β4 nAChRs was 90% blocked by 10 μM α-EI [28].

**Table 3:** Peptides of fish-hunting *Conus* that block nAChRs.^1.^ Cysteine are in bold

Peptide ^2^	Sequence ^3^	Reference
α(3/5)		
α-GI	E**CC**NPA**C**GRHYS**C**#	Gray 1981 [37]
α-GIA	E**CC**NPA**C**GRHYS**C**GK#	Gray 1981 [37]
α-GIB	E**CC**NPA**C**GRHYS**C**KG#	McIntosh 2002 [40]
α-GII	E**CC**HPA**C**GKHFS**C**#	Gray 1981 [37]
α-MI	GR**CC**HPA**C**GKNYS**C**#	McIntosh 1982 [8]
α-MIA	DGR**CC**HPA**C**AKHFN**C**#	McIntosh 2002 [40]
α-MIB	NGR**CC**HPA**C**GKNYS**C**#	McIntosh 2002 [40]
α-MIC	**CC**HPA**C**GKNYS**C**#	Kapono 2013 [39]
α-SI	I**CC**NPA**C**GPKYS**C**#	Zafaralla 1988 [41]
α-SIA	Y**CC**HPA**C**GKNFD**C**#	Myers 1991 [42]
α-SII	G**CCC**NPA**C**GPNYG**C**GTS**C**S	Ramilo 1992 [43]
α-Ac1.1a	NGR**CC**HPA**C**GKHFN**C**#	Liu 2007 [44]
α-Ac1.1b	NGR**CC**HPA**C**GKHFS**C**#	Liu 2007 [44]
α-CnIA	GR**CC**HPA**C**GKYYS**C**#	Favreau 1999 [45]
α-CnIB	**CC**HPA**C**GKYYS**C**#	Favreau 1999 [45]
α(4/7)		
α-EI	RDO**CC**YHPT**C**NMSNPQI**C**#	Martinez 1995 [22]
α-MII†	G**CC**SNPV**C**HLEHSNL**C**#	Cartier 1996 [9]
α-GIC†	G**CC**SHPA**C**AGNNQHI**C**#	McIntosh 2002 [40]
α-GID†	IRDγ**CC**SNPA**C**RVNNOHV**C**	Nicke 2003 [46]
α-PIA†	RDP**CC**SNPV**C**TVHNPQI**C**#	Dowell 2003 [47]
α-PeIA†	G**CC**SHPA**C**SVNHPEL**C**#	McIntosh 2005 [48]
α(4/4)		
α-BuIA†	G**CC**STPP**C**AVLY**C**#	Azam 2005 [49]
α-PIB	ZSOG**CC**WNPA**C**VKNR**C**#	Lopez-Vera 2007 [50]
α-EIIA	ZTOG**CC**WNPA**C**VKNR**C**#	Quinton 2013 [51]
αA_Long_		
αA-PIVA	G**CC**GSYONAA**C**HO**C**S**C**KDROSY**C**GQ#	Hopkins 1995 [52]
αA-EIVA	G**CC**GPYONAA**C**HO**C**G**C**KVGROOY**C**DROSGG#	Jacobsen 1997 [33]
αA-EIVB	G**CC**GKYONAA**C**HO**C**G**C**TVGROOY**C**DROSGG#	Jacobsen 1997 [33]
αA_Short_		
αA-OIVA	**CC**GVONAA**C**HO**C**V**C**KNT**C**#	Teichert 2004 [53]
αA-OIVB	**CC**GVONAA**C**PO**C**V**C**NKT**C**G#	Teichert 2005b [54]
αA-PeIVA	**CC**GVONAA**C**HO**C**V**C**TGK**C**	Teichert 2006 [55]
αS		
αS-RVIIA	K**C**NFDK**C**KGTGVYN**C**GγS**C**S**C**γGLHS**C**R**C**TY	
	NIGSMKSG**C**A**C**I**C**TYY	Teichert 2005a [54]
αS-GVIIIB†	SGST**C**T**C**FTSTN**C**QGS**C**E**C**LSPPG**C**Y**C**SNN	
	GIRQRG**C**S**C**T**C**PGT#	Christensen 2015 [56]
αC		
αC-PrXA	TYGIYDAKPOFS**C**AGLRGG**C**VLPONLROKFKE#	Jimenez 2007 [29]
αM		
ψ-PIIIE	HOO**CC**LYGK**C**RRYOG**C**SSAS**CC**QR#	Shon 1997 [20]
ψ-PIIIF	GOO**CC**LYGS**C**ROFOG**C**YNAL**CC**RK#	Van Wagoner 2003 [57]
ψ-Pr3e	AAR**CC**TYHGS**C**LKEK**C**RRKY**CC**#	Lluisma 2008 [18]
αM-MIIIJ	ZK**CC**SGGS**C**PLYFRDRLI**C**P**CC**	This report

^1^ All peptides listed block muscle nAChRs except for the following seven that are tagged with a dagger symbol (†): α-MII, α-GIC, α-GID, α-PIA, α-PeIA, α-BuIA, and αS-GVIIIB, which preferentially block neuronal nAChRs over muscle nAChRs for exogenously expressed mammalian receptors (e.g., α6β3β2 and α3β2 for α-MII; α3β2 for α-GIC, and α6β3β2 for α-PIA). ^2^ First letter following the hyphen indicates *Conus* species, as follows: Ac, *achatinus*; E, *ermineus*; G, *geographus*; M, *magus*; O, *obscurus*; P, *purpurascens*; Pe, *pergrandis*; Pr, *parius*; R, *radiatus*; and S, *striatus*. ^#^ indicates C-terminal amidation; O, hydroxyproline; Z, pyroglutamate; γ, gamma-carboxyglutamate.

## Supplementary Materials

The following are available online at https://www.mdpi.com/2072-6651/12/3/197/s1, Figure S1: HPLC elution profile of *C. magus* venom. Figure S2: αM-MIIIJ (10 μM) inhibits fluorescently-tagged α-BgTX binding at neuromuscular junctions of goldfish intercostal muscles, Figure S3: αM-MIIIJ (10 μM) inhibits binding of fluorescently-tagged α-BgTX at both septal and distributed neuromuscular junctions in zebrafish, Table S1: Paralysis of goldfish induced by intramuscular injection of αM-MIIIJ.
