# Supplementary material for: αM-Conotoxin MIIIJ Blocks Nicotinic Acetylcholine Receptors at Neuromuscular Junctions of Frog and Fish
Source: Toxins (Basel). 2020 Mar 21;12(3):197. doi: 10.3390/toxins12030197 (PMC7150935; doi:10.3390/toxins12030197)
Supplement: Supplementary file 1 [file toxins-12-00197-s001.pdf]

Supplementary Materials: αM-Conotoxin MIIIJ Blocks Nicotinic Acetylcholine Receptors at Neuromuscular Junctions of Frog and Fish

Matthew J. Rybin, Henrik O'Brien, Iris Bea L. Ramiro, Layla Azam, J. Michael McIntosh, Baldomero M. Olivera, Helena Safavi-Hemami * and Doju Yoshikami *

Table S1. Paralysis of goldfish induced by intramuscular injection of αM-MIIIJ.[1.]

αM-MIIIJ Injected (nmol)	Weight[2] (g)	Time to Paralysis (min:sec)
0.5	1.186	7:17
	0.752	23:47
1.5	1.596	8:12
	1.016	1:27
	1.108	11:23
5.0	1.394	2:55
	1.682	1:30
	1.338	2:22

[1]Injections and behavioral observations were performed as described in Methods. [2]Weight of injected fish.

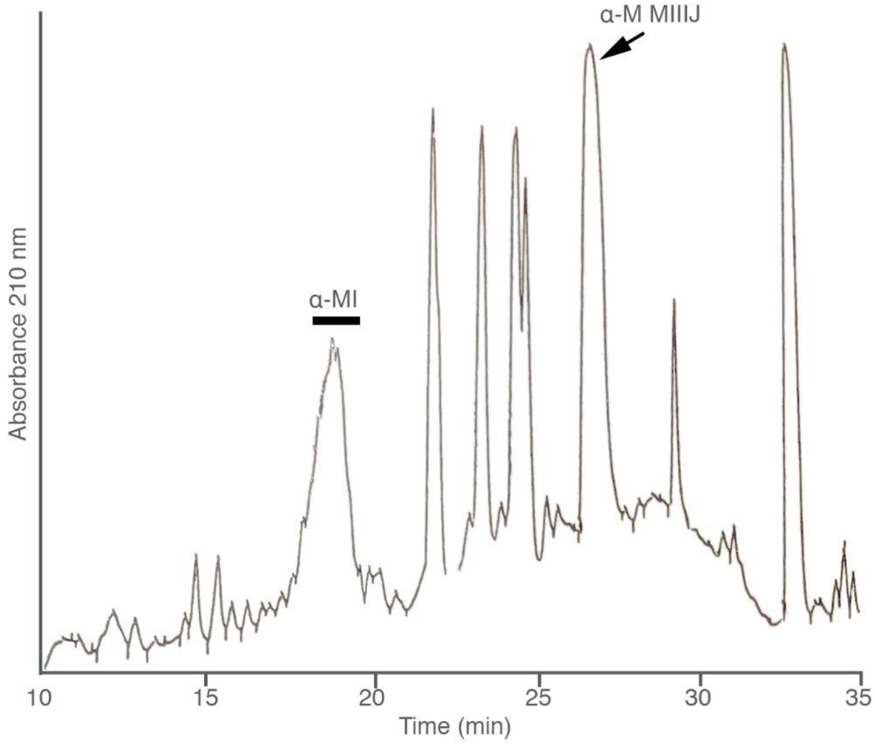

Figure S1. HPLC elution profile of *C. magus* venom. Chromatography was performed as described in Methods and as previously published for α-MI [8]. αM-MIIIJ eluted at peak indicated by an arrow; for reference, bar shows location where α-MI eluted.

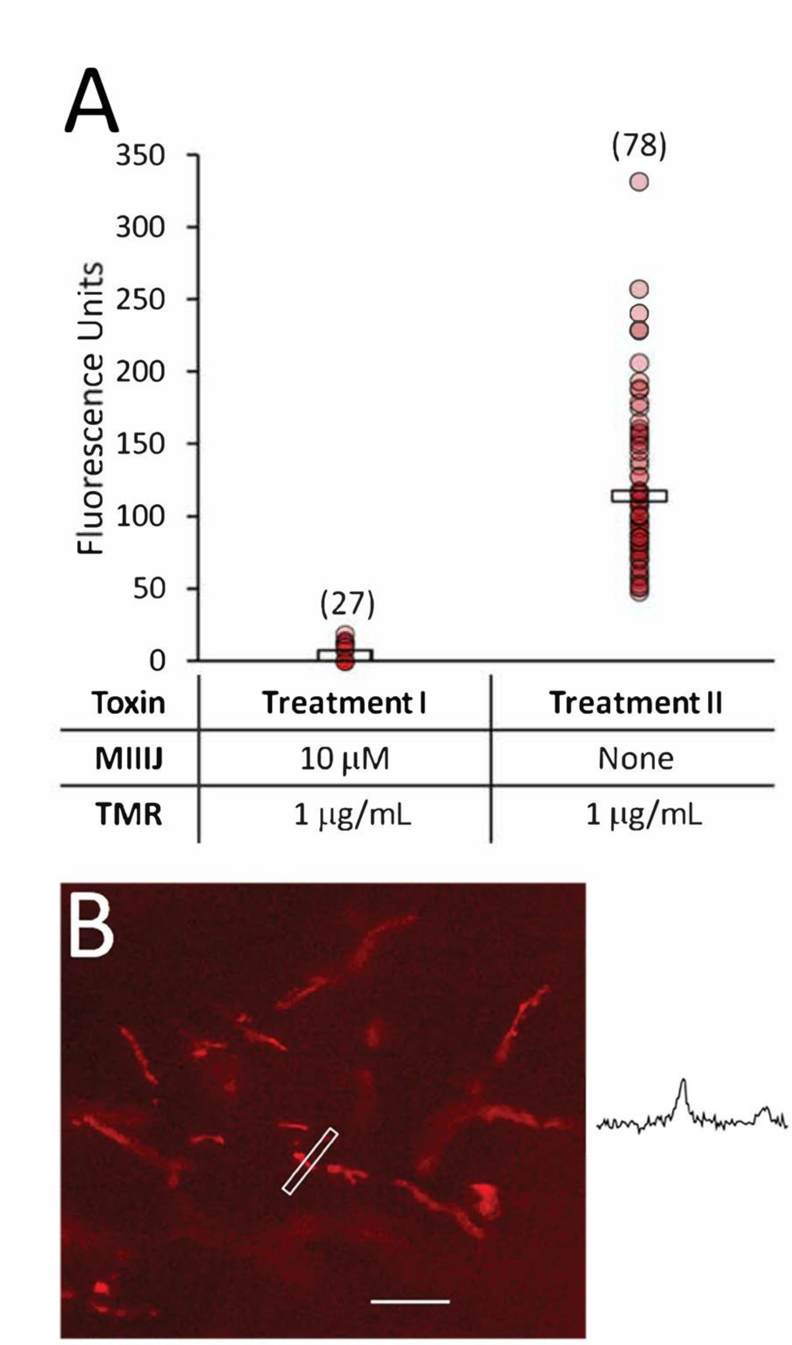

Figure S2. αM-MIIIJ (10 μM) inhibits fluorescently-tagged α-BgTX binding at neuromuscular junctions of goldfish intercostal muscles. Muscle preparation and imaging were performed as described in Methods. **A.** Fluorescence-intensity measurements following 10-min. exposure to 1 μg/mL TMR α-BgTX in the presence (Treatment I) or absence (Treatment II) of 10 μM αM-MIIIJ, followed by washing. Data are from four muscle preparations, two for each treatment. Each data point represents one measurement, and horizontal bar represents mean value of the points from each treatment. Number of data points from each treatment is shown in parentheses. The data points from Treatment II all lie above those from Treatment I, clearly indicating that α-BgTX binding was inhibited by αM-MIIIJ. **B.** Representative fluorescence image of a muscle subjected to Treatment II, with fluorescence profile along ROI (rectangle) plotted at right. Calibration bar in image represents 20 μm.

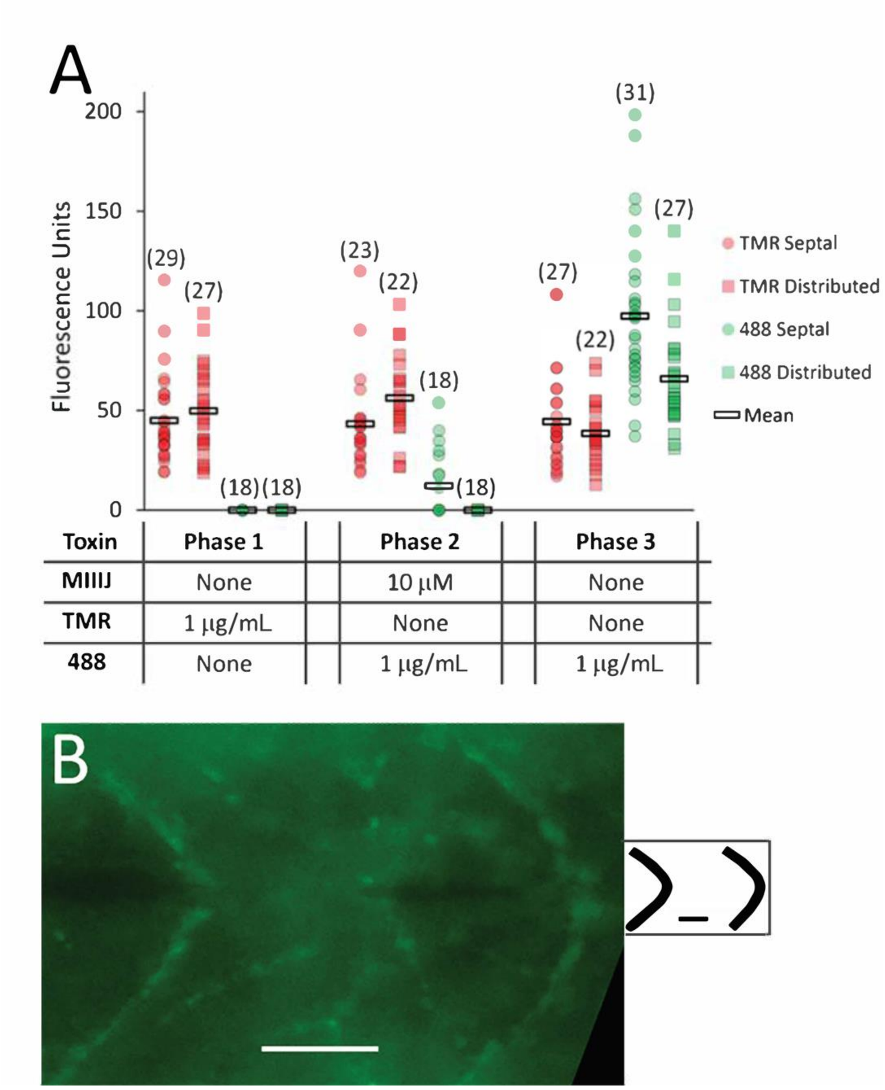

Figure S3. αM-MIIIJ (10 µM) inhibits binding of fluorescently-tagged α-BgTX at both septal and distributed neuromuscular junctions in zebrafish. Muscles of larval zebrafish were prepared and imaged as described in Methods. **A.** Red and green fluorescence intensities of muscle preparations from four fish, first stained with TMR α-BgTX (1 µg/mL for 10 min) then rinsed and imaged (Phase 1); then, following a 10-min pre-treatment with 10 µM αM-MIIIJ, stained for 10 min with Alexa488 α-BgTX (1 µg/mL) in the presence of 10 µM αM-MIIIJ then rinsed and imaged (Phase 2); and lastly, stained for 10 min with Alexa488 α-BgTX alone (1 µg/mL) then rinsed and imaged (Phase 3). Values of fluorescence at myosepta (i.e., presumably of slow fibers) are represented by circles (colored red for TMR and green for Alexa488) while fluorescence at NMJs distributed elsewhere (i.e., presumably of fast fibers) are represented by squares (red for TMR and green for Alexa488). Each data point represents a measurement, and the average value for a given column is represented by the open horizontal rectangle. Number in parentheses indicates the number of data points in each column. TMR-fluorescence values for fast and slow fibers were similar and remained essentially the same (~50 Fluorescence Units) in all phases showing that AChR receptor densities at both synapses of both fiber types are similar and TMR α-BgTX remained bound throughout the experiment. The lower mean values of green fluorescence in Phase 2 compared to those in Phase 3 show that αM-MIIIJ inhibited Alexa488 α-BgTX binding at both fast and slow fibers. **B.** Image of zebrafish muscle preparation treated with Alexa488 α-BgTX illustrating the two staining patterns nAChRs: chevron-shaped septa staining representative of slow fibers, and distributed punctate staining of fast fibers. Shown at right is a ¼-scale cartoon depicting locations of the two septa in image. Calibration bar in image and sketch, 20 µm.
